# Characterization of genetic aberrations in a single case of metastatic thymic adenocarcinoma

**DOI:** 10.1186/s12885-017-3282-9

**Published:** 2017-05-15

**Authors:** Yeonghun Lee, Sehhoon Park, Se-Hoon Lee, Hyunju Lee

**Affiliations:** 1 School of Electrical Engineering and Computer Science, Gwangju Institute of Science and Technology, 123 Cheomdangwagi-ro, Buk-gu, Gwangju, 61005 South Korea; 2 Division of Hematology and Medical Oncology, Department of Internal Medicine, Seoul National University Hospital, 101 Daehakro, Jongnogu, Seoul, 110-744 South Korea; 3 Division of Hematology/Oncology, Department of Medicine, Samsung Medical Center, Sungkyunkwan University School of Medicine, 81 Irwon-Ro Gangnam-gu, Seoul, 06351 South Korea

**Keywords:** Thymic adenocarcinoma, Thymic epithelial tumors, Whole exome sequencing, Somatic mutations, Somatic copy number alterations

## Abstract

**Background:**

Thymic adenocarcinoma is an extremely rare subtype of thymic epithelial tumors. Due to its rarity, there is currently no sequencing approach for thymic adenocarcinoma.

**Methods:**

We performed whole exome and transcriptome sequencing on a case of thymic adenocarcinoma and performed subsequent validation using Sanger sequencing.

**Results:**

The case of thymic adenocarcinoma showed aggressive behaviors with systemic bone metastases. We identified a high incidence of genetic aberrations, which included somatic mutations in RNASEL, PEG10, TNFSF15, TP53, TGFB2, and FAT1. Copy number analysis revealed a complex chromosomal rearrangement of chromosome 8, which resulted in gene fusion between MCM4 and SNTB1 and dramatic amplification of MYC and NDRG1. Focal deletion was detected at human leukocyte antigen (HLA) class II alleles, which was previously observed in thymic epithelial tumors. We further investigated fusion transcripts using RNA-seq data and found an intergenic splicing event between the CTBS and GNG5 transcript. Finally, enrichment analysis using all the variants represented the immune system dysfunction in thymic adenocarcinoma.

**Conclusion:**

Thymic adenocarcinoma shows highly malignant characteristics with alterations in several cancer-related genes.

**Electronic supplementary material:**

The online version of this article (doi:10.1186/s12885-017-3282-9) contains supplementary material, which is available to authorized users.

## Background

Thymic adenocarcinoma is an extremely rare subtype of thymic carcinoma. Thirty-five cases have been reported in the literature since the first case in 1989 [1–5]. They have shown papillary, papillotubular, tubular, or mucinous histological features that were different from the other subtypes of thymic carcinoma. Immunohistochemically, thymic adenocarcinomas have been distinguished from other metastatic adenocarcinomas in the anterior mediastinum [6, 7].

With their distinct entities, thymic adenocarcinomas have shown higher malignancy than other thymic epithelial tumors (TETs). They exhibited rapid metastasis, frequently infiltrating the superior vena cava, pleura and pericardium [8–10]. Fifteen cases (42.86%) of thymic adenocarcinoma showed metastatic lesions to adjacent organs, including lung (31.43%), lymph node (22.86%), bone (11.43%), and liver (5.71%) [3, 4, 8–17]. Most cases of thymic adenocarcinoma were resistant to chemotherapy and radiotherapy with poor prognosis. Of 19 cases resected surgically [2, 4, 11, 13, 16–19], multiple progressive metastases developed in six cases [4, 11, 13, 16].

Their exceptional occurrences and clinical challenges have triggered investigations into genetic aberrations in thymic adenocarcinoma. Recently, a case of thymic adenocarcinoma showed the deletion of the HLA-DRB5 locus on array comparative genomic hybridization (CGH) analysis [2]. Genomic or transcriptional losses of HLA class II alleles have been recurrent in TETs [20, 21], although the association between HLA class II alleles and TETs is still controversial because they are highly polymorphic. Until now, other putative driver mutations except the deletion of the HLA-DRB5 locus have not been discovered yet in thymic adenocarcinoma. Thus, for the first time, we performed the whole exome sequencing (WES) and whole transcriptome sequencing (WTS) for genetic understanding of thymic adenocarcinoma.

## Methods

### Sample preparation

A fresh-frozen tissue sample from a 31-year-old man with thymic adenocarcinoma was acquired by gun-biopsy. This study was approved by the Institutional Review Board (IRB) of Seoul National University Hospital (1206–086-414). The patient provided his written informed consent to participate in this study. The informed consent form included the publication of his clinical details and/or clinical images. Genomic DNA and total RNA were extracted by using the QIAamp DNA Micro Kit (Qiagen, Valencia, CA, USA) and PureLink RNA mini kit (ambion), respectively. Genomic DNA from the peripheral blood of the patient was used as a matched normal, which was extracted by the Maxwell 16 LEV Blood DNA Kit (Promega, Madison, WI, USA).

### Sequencing (WES and WTS)

Genomic DNA was randomly fragmented (250-300 bp fragments) and amplified by ligation-mediated polymerase chain reaction (PCR). For WES, DNA was captured by the SureSelectXT Human All Exon V4 + UTR 71 Mb kit (Agilent Technologies, Santa Clara, CA, USA). Libraries for WTS were prepared by the TruSeq RNA Sample Preparation kit (Illumina, San Diego, CA, USA). WES and WTS were performed by the Illumina Hiseq2000 platform (Illumina) and generated 101 bp pair-end reads.

### Detection of somatic SNVs and indels

Varscan2 [22] and Mutect [23] were used to identify somatic mutations from WES data. First, we obtained the raw output of somatic single nucleotide variants (SNVs) and indels using Varscan2, and then applied the high-confidence and false-positive filters with options of a variant frequency >=10% and variant read counts >4. In addition, variants in base repeats greater than five were filtered out. Second, we used Mutect to find somatic SNVs and false-positive SNVs were removed by the internal variant filter. Third, we selected somatic SNVs that were detected by both Varscan2 and Mutect while somatic indels were selected independently from Varscan2.

Next, we selected nonsynonymous variants within exonic, splicing and ncRNA exonic regions. All the variants were annotated by ANNOVAR [24]. To prioritize functional variants, mutations within a non-conserved region were filtered out (phastConsElements46way). Common single nucleotide polymorphisms (SNPs) were removed by annotating in 1000 Genomes Projects and dbSNP. False positives by paralogous alignments were filtered out using genomicSuperDups. Lastly, cancer census mutations were annotated using COSMIC (cosmic68).

### Copy number analysis

We utilized two pipelines for copy number analysis using WES data: Varscan2 followed by a circular binary segmentation (CBS) [25] and EXCACVATOR [26]. From Varscan2, we obtained raw bins with a size of 50 - 200 bp, which were delimited by CBS with options of alpha = 0.01, nperm = 10,000, and undo.SD = 3. Next, we applied an additional merging step in which we first excluded focal noises predicted by fewer than 25 copy number bins and then we iteratively merged adjacent segments within the copy number difference < 0.2. For EXCAVATOR, we made targeted regions by merging all the probes of the SureSelectXT Human All Exon V4 + UTR 71 Mb kit. Targeted regions with less than 30X coverage were excluded. We called the HSLM algorithm with options of 1) Omega = 0.1; 2) Theta = 1e-3; and 3) D_norm parameter = 10e6. Segments supported by fewer than ten exons were filtered out.

After the initial segmentation steps of Varscan2 and EXCAVATOR, we classified segments into large-scale somatic copy number alterations (SCNAs; >25% of a chromosome arm) and focal SCNAs (<=25% of a chromosome arm). We used copy number thresholds of −0.25 and 0.3 for large-scale SCNAs and −0.8 and 0.9 for focal SCNAs. We only selected large-scale SCNAs and focal SCNAs (>=10 exons) that were detected by both pipelines. Focal SCNAs (<10 exons) were just estimated using the Varscan2 pipeline because EXCAVATOR has a lower sensitivity for regions with a small number of exons. The 3′ UTR-biased discoveries with a high fraction (>60%) of copy number bins in a 3′ UTR region which was not properly targeted by WES were further filtered out.

### Detection of fusion genes using WES and WTS

Gene fusion analysis using WES data was performed on regions captured by the SureSelectXT Human All Exon V4 + UTR 71 Mb kit (>30X coverage). We used FACTERA [27] for inter-gene fusions with options of minimum split reads = 30 and minimum discordant reads = 10. We removed fusion events located in repeated regions from RepeatMasker and GenomicSuperDups. To detect fusion transcripts, we used defuse [28] with default options and filtered out fusion events supported by fewer than 50 split reads. Transcript fusion events located in repeated regions were also excluded. The read-through event was further confirmed by using MapSplice [29].

### Sanger sequencing validation

We validated seven somatic mutations (six SNVs and a small insertion) and a gene fusion event via Sanger sequencing. Genomic positions and designed primers were summarized in Additional file 1: Table S1. PCR was implemented using h-Taq DNA polymerase (genes with somatic mutations) and Dr. MAX DNA polymerase (the fusion gene) on DNA Engine Tetrad 2 Peltier Thermal Cycler. PCR products were purified and prepared for Sanger sequencing with the BigDye Terminator v3.1 Cycle Sequencing Kit. They were sequenced by the ABI PRISM 3730XL Analyzer.

## Results

### Clinical and pathological findings

**Fig. 1:**
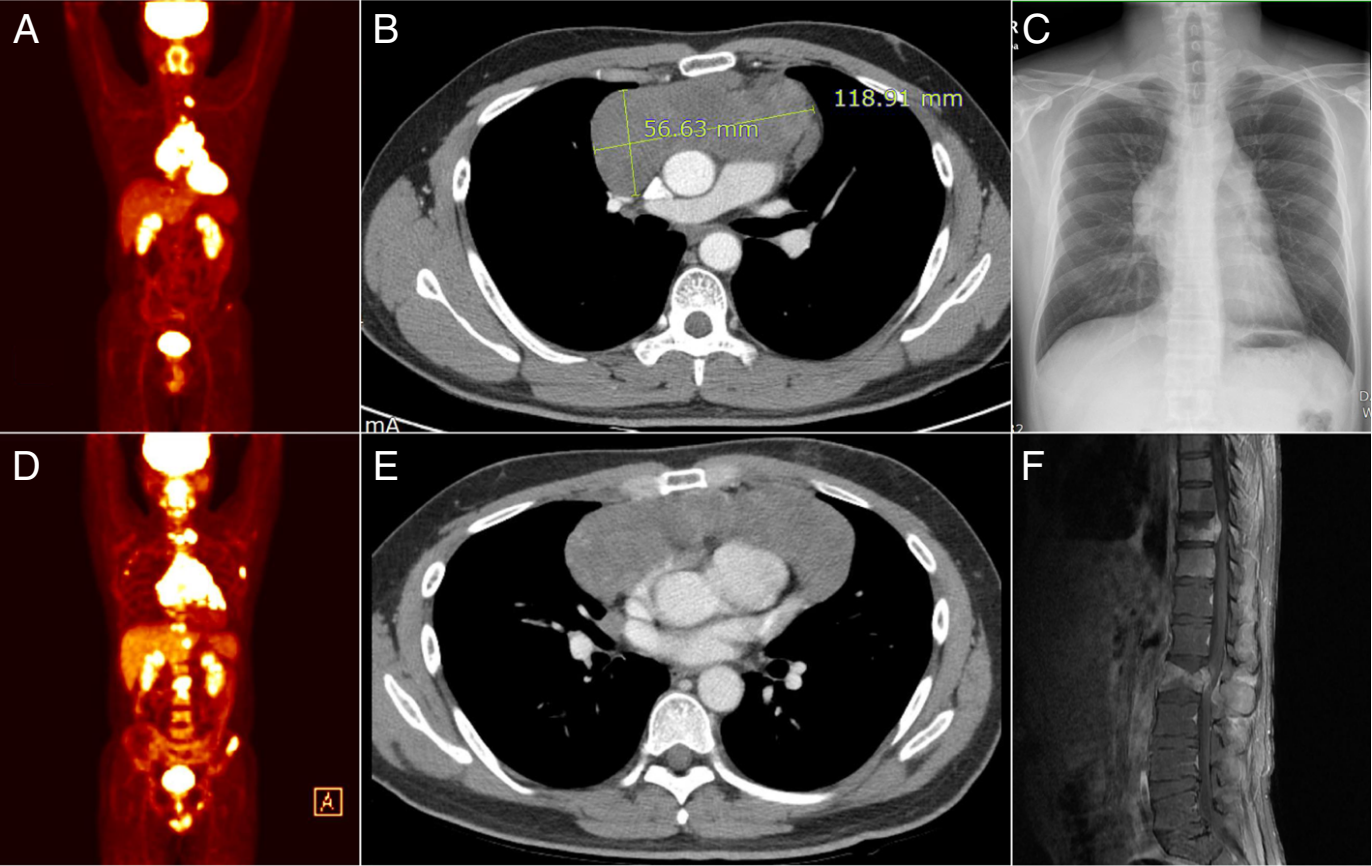
Radiologic presentation of thymic adenocarcinoma and systemic metastases. Top images **a**, **b**, and **c** represent the initial presentation of thymic adenocarcinoma. Bottom images **d**, **e**, and **f** indicate disease progression observed 3 to 5 months after the initial workup. **a** PET-CT scan shows the large mass in the anterior mediastinum with metastatic lesions. **b** Chest CT shows 118.91 × 56.63 mm sized mass with focal calcification. **c** Chest X-ray shows the anterior mediastinal mass. **d** PET-CT scan after 3 months reveals metastatic lesions to mediastinal lymph nodes and multiple bones. **e** Chest CT after 4 months reveals the enlarged mass and metastatic lung nodules. **f** Spine MRI after 5 months reveals the significant cord compression at T10 and severe central canal stenosis at L2

A 31-year-old man presented with chest tightness and discomfort for two weeks’ duration. Positron emission tomography/computed tomography (PET/CT) revealed an anterior mediastinal mass with metastatic lesions at the left supraclavicular lymph node and pelvic bone (Fig. 1a). Chest X-ray and CT showed the 11.9 cm × 5.7 cm main mass occupying the anterior mediastinum (Fig. 1b, c). Serum levels of CEA and CA19–9 were elevated (37.5 ng/ml and 64.9 U/ml, respectively), although AFP and beta-hCG were normal. Abdominal CT additionally excluded an upper gastrointestinal origin. He was treated with a combination chemotherapy, which included doxorubicin, cyclophosphamide, and cisplatin. And then he was treated with another combination regimen consisting of etoposide, ifosfamide, and cisplatin. After three months since the start of first treatment, systemic metastases were developed in the thoracic and lumbar spine, mediastinal lymph nodes, and pelvis (Fig. 1d). The main mass was enlarged with metastatic lung nodules (Fig. 1e). The patient received laminectomy because of the significant cord compression by tumor extension at the T10 and L2 vertebrae (Fig. 1f). CD117 (c-kit) staining was positive in metastatic bone tumors, indicating metastasis from the thymic carcinoma origin.

### Histological and immunohistochemical (IHC) findings

**Fig. 2:**
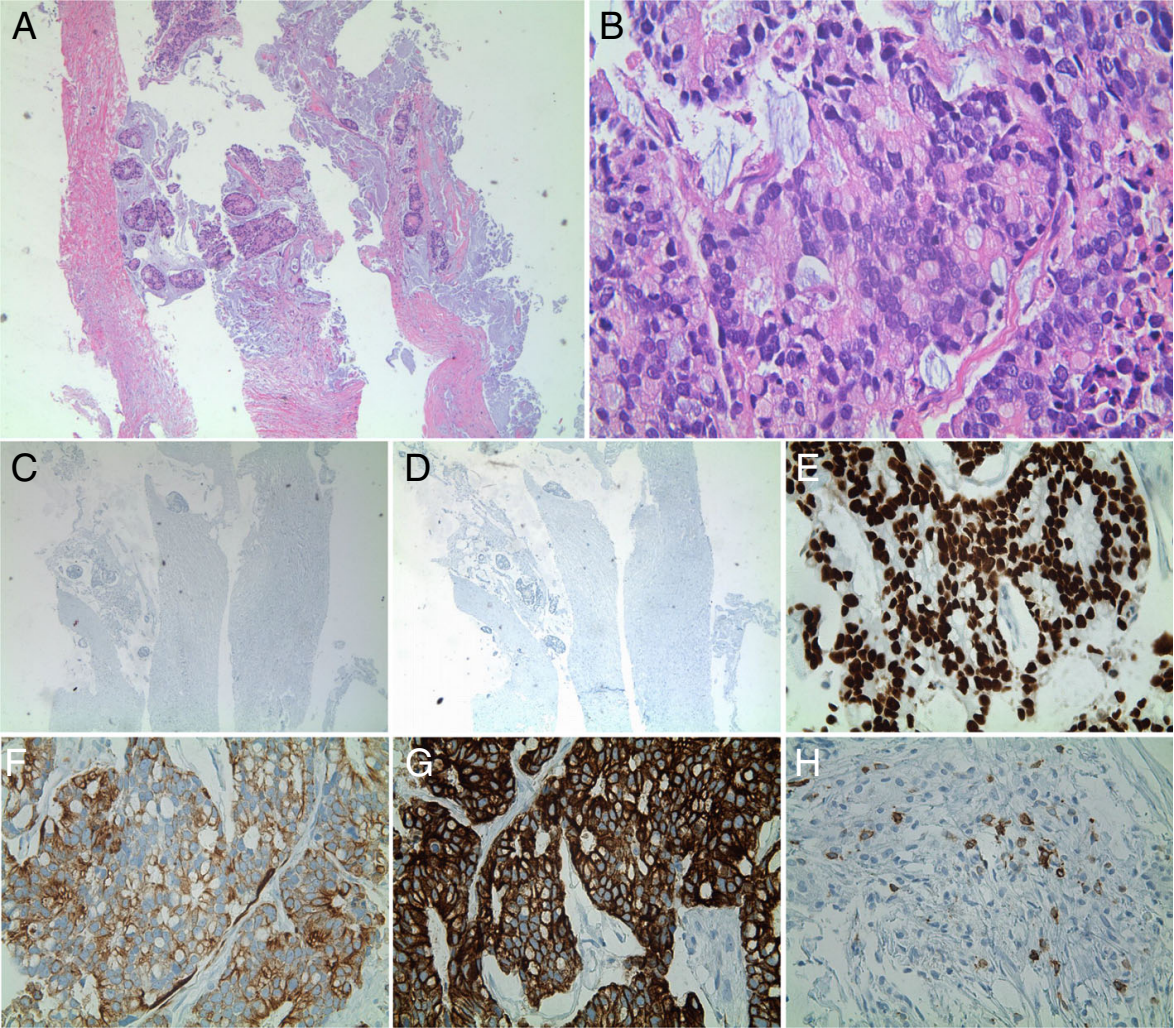
Representative images of H&E and IHC staining of thymic adenocarcinoma. **a** Hematoxylin and eosin (H&E) staining of the soft tissue specimen from the needle biopsy shows the involvement of carcinoma with extracellular mucin pools (×40) and **b** mucin-producing epithelial cells (×400). **c** IHC staining shows negativity for TTF1 and **d** HER2, but positivity for **e** CDX2, **f** CK7, and **g** CK20. **h** Focal positivity for CD5 is shown in lymphoid cells (×400)

A needle biopsy confirmed the involvement of carcinoma with extracellular mucin pools and mucin-producing epithelial cells. (Fig. 2a, b). IHC staining was negative for TTF1 and HER2 (Fig. 2c, d), but positive for CDX2, CK7, and CK20 (Fig. 2e–g). Focal positivity for CD5 was shown in lymphoid cells (Fig. 2h). These histological features and IHC staining patterns were consistent with previous reports of thymic mucinous adenocarcinoma.

### Somatic mutations

From WES data of the patient, we detected total 55 somatic mutations in exonic regions and 23 mutations of them were synonymous SNVs (Additional file 2: Table S2). Functional mutations were prioritized by several reducing processes (see Methods). As a result, we obtained 19 SNVs and a 6-base pair insertion (Table 1). The 6-base pair insertion was found in FAT1. FAT1 also harbored a somatic SNV (G > A) near the 6-base pair insertion in the same allele (Additional file 3: Figure S1). FAT1 is a tumor suppressor gene involved in the Wnt signaling, and FAT1 inactivation has been reported to promote tumor growth [30]. Next, we investigated WTS data to find transcribed variants among 20 somatic variants. WTS supported seven somatic variants (APH1A, RNASEL, TNFSF15, NOL6, TNEM3, GZF1, and TP53), which showed similar allele frequencies between WES and WTS (Table 1). We validated six cancer-related genes (RNASEL, PEG10, TNFSF15, TP53, TGFB2, and FAT1) using Sanger sequencing (Additional file 4: Figure S2).

**Table 1:** Somatic SNVs and a small insertion detected by WES and WTS

			Sequence			Allele frequency	
Gene	Chr	Position	Ref	Var	Coding change	WES	WTS
APH1A	chr1	150,239,812	A	T	p.L142H	41/133 (30.83%)	161/630 (25.56%)
RNASEL	chr1	182,554,694	G	C	p.H416Q	21/151 (13.91%)	2/15 (13.33%)
GPR124	chr8	37,695,290	T	A	p.S698T	26/74 (35.14%)	0/35 (0%)
WDR87	chr19	38,384,338	G	C	p.L669V	33/177 (18.64%)	0/0 (0%)
ZBTB34	chr9	129,642,990	G	A	p.G434R	37/160 (23.12%)	0/0 (0%)
PEG10	chr7	94,293,260	G	A	p.R207H	37/193 (19.17%)	0/2 (0%)
TNFSF15	chr9	117,552,937	A	G	p.V184A	17/112 (15.18%)	2/2 (100%)
NOL6	chr9	33,464,880	T	A	p.T926S	16/89 (17.98%)	20/208 (9.61%)
ONECUT1	chr15	53,082,000	G	T	p.L28M	21/47 (44.68%)	0/0 (0%)
CASKIN1	chr16	2,229,210	A	T	p.S1298T	4/15 (26.67%)	0/4 (0%)
A3GALT2	chr1	33,772,594	G	A	p.R266C	7/36 (19.44%)	0/0 (0%)
TENM3	chr4	183,721,259	C	T	p.R2619C	17/61 (27.87%)	1/3 (33.33%)
GZF1	chr20	23,346,065	C	T	p.R349C	21/102 (20.59%)	2/9 (22.22%)
MAFA	chr8	144,512,176	G	A	p.T134M	11/44 (25%)	0/3 (0%)
TP53	chr17	7,578,403	C	A	p.C176F	51/91 (57.14%)	44/84 (52.38%)
TGFB2	chr1	218,607,460	C	T	p.R211C	10/64 (15.62%)	0/2 (0%)
SEL1L2	chr20	13,856,683	C	T	splice donor variant	30/120 (25%)	0/0 (0%)
SPTA1	chr1	158,627,313	G	C	p.P920R	27/99 (27.27%)	0/0 (0%)
FAT1	chr4	187,527,274	T	+GACATC	nonframeshift	14/51 (27.45%)	0/0(0%)
FAT1	chr4	187,527,282	G	A	p.S3431F	14/51 (27.45%)	0/0(0%)

A C > A substitution encoding p.C176F in TP53 was detected with the highest allele frequency. The p.C176F mutation of TP53 was previously reported in the COSMIC database [31], which showed high occurrences of the p.C176F variant in thoracic tumors such as lung, esophagus, and upper aerodigestive tract (Additional file 5: Table S3). Besides the TP53 variant, an A > G substitution (p.V184A) and C > T substitution (p.R211C) were found in TNFSF15 and TGFB2, respectively, which are cancer-related immunocytokine genes. TNFSF15 encodes TNF-like ligand 1A (TL1A), which costimulates T cells with highly regulated expression [32]. TGFB2 is a member of the TGFB family, which has tumor promoting functions enhancing the epithelial-mesenchymal transition and evasion of immunity [33]. Especially, the p.R211C mutation occurred in the propeptide domain of TGFB2 (Additional file 6: Figure S3), which is responsible for maintaining the latency of TGFB2 [34]. Point mutations (R218C, C223R, and C225R) in the propeptide of TGFB1 have been reported to increase the active TGFB1 level [35, 36]. Similarly, the p.R211C mutation of TGFB2 might affect the latency of TGFB2.

### Large-scale SCNAs

We analyzed SCNAs using Varscan2 and EXCAVATOR (see Methods) and identified 14 large-scale SCNAs, which included four large-scale amplification (chr5p, chr12p, chr7, and chr20) and 10 large-scale deletions (chr3p, chr4p, chr6q, chr8p, chr9p, chr10p, chr15q, chr17p, chr21q, and chr16). Especially, chr4p, chr8p, and chr10p deletions and chr12p amplifications were noticeable (log2 ratio < −0.75 and > 0.75 each). Thymic carcinomas have shown more frequent arm-level SCNAs than thymomas [37, 38]. Similarly, thymic adenocarcinoma showed a considerable number of arm-level SCNAs.

### Complex chromosomal rearrangements of chromosome 8 with MYC amplification

**Fig. 3:**
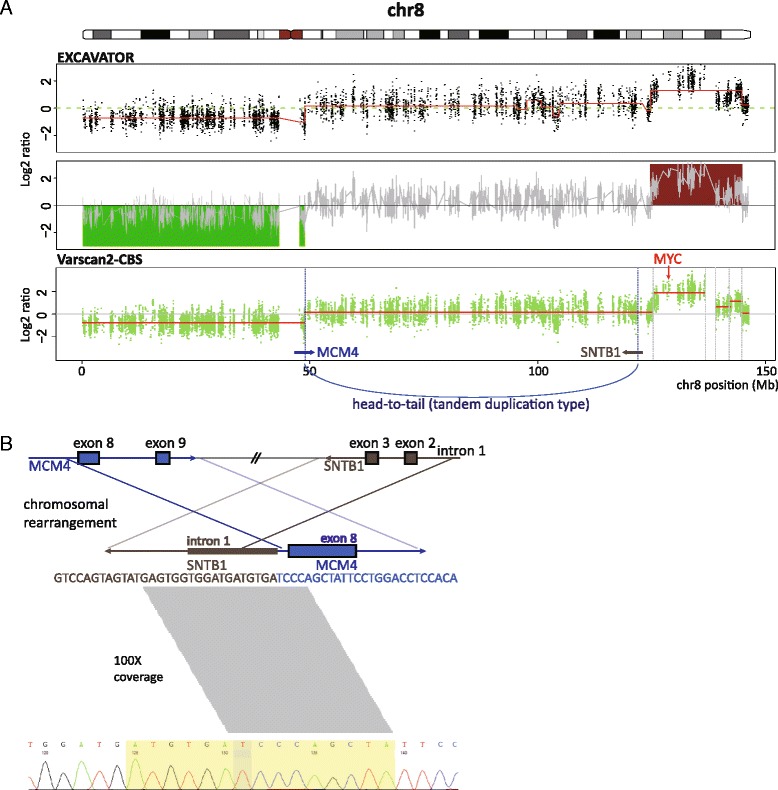
Complex chromosomal rearrangements of chromosome 8 with MYC amplification. **a** SCNAs of chromosome 8 detected from WES data. EXCAVATOR and Varscan2-CBS discover deletion of the whole chr8p and focal amplification of the end of chr8q, which encompasses MYC. **b** The gene fusion event between MCM4 and SNTB1. Exon 8 of MCM4 and intron 1 of SNTB1 are fused by the intrachromosomal rearrangement

Copy number analysis uncovered complex copy number states of chromosome 8 (Fig. 3a). With the loss of the whole p arm of chromosome 8 (log2 ratio = −0.77), the end of the q arm showed stepwise copy number increases. The most highly amplified region (log2 ratio = 1.89) included NDRG1 and MYC. With complex copy number states, an intrachromosomal rearrangement (head-to-tail, tandem duplication type) was found between MCM4 and SNTB1 (Additional file 7: Table S4). Breakpoints of the head-to-tail fusion (chr8:48,878,682 and 121,816,370) were found coincidently near copy number breakpoints (chr8:48,878,526 and 125,164,614) (Fig. 3a). The head-to-tail fusion with loss of chr8p possibly implied monosomy 8 with duplicated chr8q arms. The fusion sequence between MCM4 exon 8 and SNTB1 intron 1 was derived from the head-to-tail fusion (Fig. 3b). The Integrative Genomics Viewer (IGV) [39] showed more than 50 clipped reads at each SV breakpoint exclusively in the tumor sample (Additional file 8: Figure S4), which indicates that the fusion sequence was newly derived in the tumor and attained more than 100X coverage. The fusion sequence was further validated by Sanger sequencing (Fig. 3b). MCM4 is involved in the MCM2-MCM7 complex essential for DNA replication licensing, and a MCM4 mutation (F345I) was reported to cause mammary adenocarcinomas in mice [40]. The MCM4 fusion event may result in dysregulated DNA replication, while the function of the MCM4 fusion gene is not yet known.

### Focal SCNAs

**Fig. 4:**
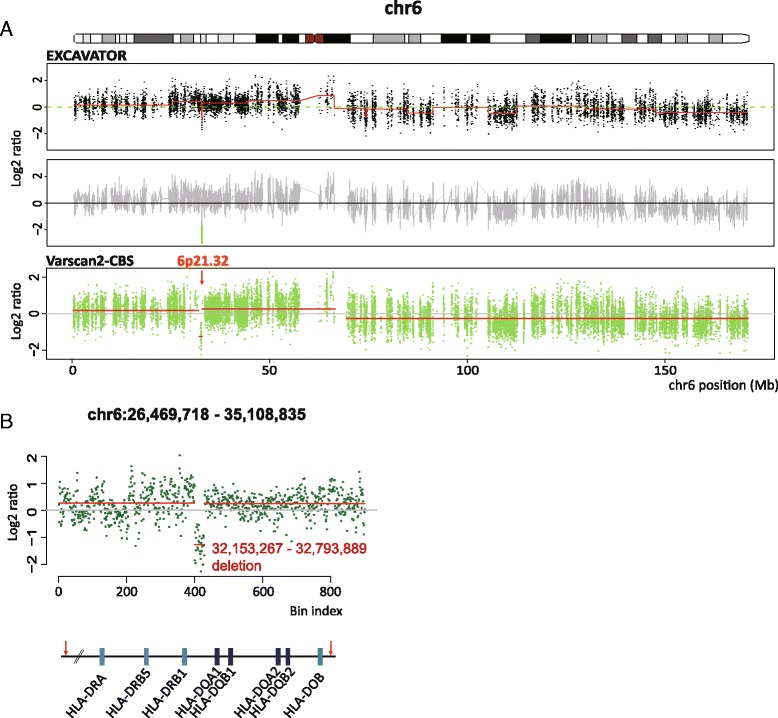
The focal deletion of chr6p21.32 encompassing HLA class II alleles. **a** The focal deletion of chr6p21.32 detected from WES data. EXCAVATOR and Varscan2-CBS consistently discover the focal deletion indicating about a log2 ratio of −1, which suggests a single-copy loss. **b** The focal segmentation result of the chromosomal region (chr6:26,469,718–35,108,835) analyzed by CBS, showing the deletion of the chromosomal region (chr6:32,153,267-32,793,889) which encompasses HLA class II alelles

We identified total five focal SCNAs (Additional file 9: Table S5). Three SCNAs out of the five focal SCNAs were detected in chr8q24.21–22, chr8q24.3, and chr6p21.32. (>10 exons). Two focal SCNAs were found in exon 1 to 5 of MUC16 and exon 6 of GPR112 (<10 exons) (Additional file 10: Figure S5). The focal deletion of chr6p21.32 was found with a slight copy number increase of chr6p (Fig. 4a). All the 29 copy number bins consecutively supported the focal deletion with a log2 ratio of −1.2664 (Fig. 4b), whose value suggests a single-copy loss. The deleted region included HLA-DR, HLA-DQ and DLA-DO alleles that encode HLA class II molecules, which are highly expressed on thymic epithelial cells in normal regulating CD4+ T cell immunity.

### Gene fusion analysis reveals the CTBS-GNG5 fusion transcript

We investigated WTS data to identify transcription-mediated fusion genes. We found two fusion transcripts: FABP2-C4orf3 and CTBS-GNG5 (Additional file 11: Table S6). The sequence of the CTBS-GNG5 fusion transcript showed a junction exactly between the end of CTBS exon 6 and the beginning of GNG5 exon 3 (Additional file 12: Figure S6). The observation suggested an intergenic splicing from the read-through transcript between CTBS and GNG5. Splice-junction alignment confirmed 76 reads derived from the fusion between CTBS exon 6 and GNG5 exon 3 (Additional file 12: Figure S6). The same fusion sequence between CTBS exon 6 and GNG5 exon 3 has been found in several cancer types and its role in the growth inhibitory function has been demonstrated [41].

### Enrichment analysis using the 39 gene set

**Fig. 5:**
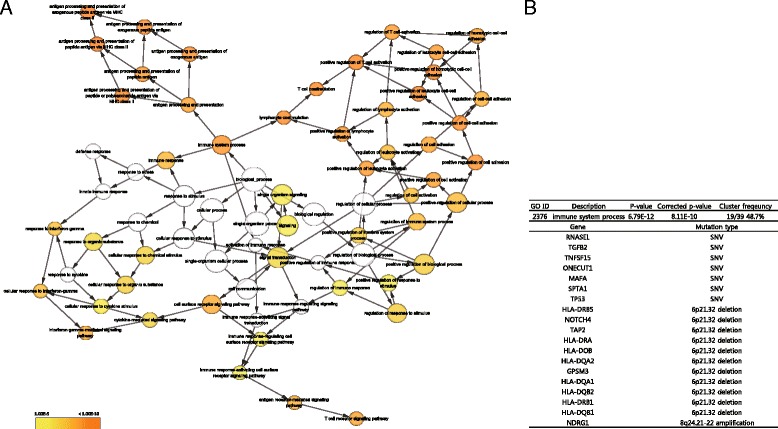
52 GO terms over-represented by the 39 gene set. **a** BiNGO analysis representing enriched 52 GO terms (*P* < 0.00001) in the biological process. **b** The gene list over-representing a GO category of the immune system process. 19 genes out of 39 genes (48.7%) that have SNVs and focal SCNAs belong to the immune system process (*P* = 8.11e-10)

For functional enrichment analysis, we integrated somatic SNVs and indels, focal SCNAs, and fusion genes. Because focal SCNAs whose sizes were larger than 1 Mbp included an excessive number of genes, we only selected cancer-census genes (MYC and NDRG1) annotated by the COSMIC database from the focal SCNAs (>1 Mbp). Consequently, we obtained a total set of 39 genes (Additional file 13: Table S7). Using BiNGO [42] (BH-corrected, *P* < 0.00001), we found 52 GO terms in the biological process (Fig. 5a and Additional file 14: Table S8). Remarkably, 19 out of 39 genes (48.7%) were annotated to the immune system process (Fig. 5b). Most of the enriched GO terms were related with T cell signaling pathways. Additionally, KEGG and Reactome enrichment analysis [43] (BH-corrected, *P* < 0.00001) showed over-representations of autoimmune diseases, chronic infections, and immunological signaling pathways (Additional file 15: Table S9).

### Genetic characteristics consistent with previous studies of thymic carcinomas

**Fig. 6:**
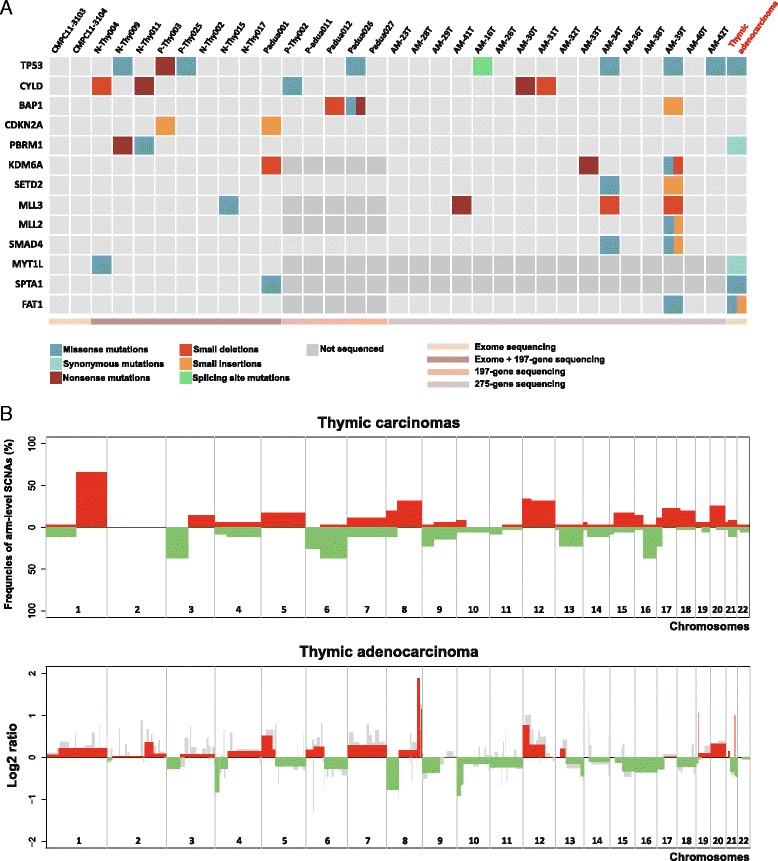
Comparisons of somatic mutations and SCNAs between thymic carcinomas and adenocarcinoma. **a** Somatic mutations that are recurrent between thymic adenocarcinoma and 32 carcinomas analyzed by exome and targeted gene sequencing. **b** Frequencies of arm-level SCNAs curated from array CGH reports of 35 carcinomas are compared with SCNAs in thymic adenocarcinoma, which are detected by Varscan2-CBS (*red and green*) and EXCAVATOR (*gray*)

A recent study reported a higher incidence of somatic mutations in thymic carcinomas than that in thymomas (average of 43.5 and 18.4, respectively) [37]. Furthermore, another study reported that thymic carcinomas exhibited more somatic mutations than thymomas in cancer-related genes, especially in TP53 [44]. The case of thymic adenocarcinoma had 55 somatic mutations including the TP53 variant. The incidence of somatic mutations seems to be consistent with previous reports of thymic carcinomas. We compared somatic mutations in thymic adenocarcinoma with somatic mutations previously discovered in 32 thymic carcinomas (Fig. 6a) [37, 45]. Five genes (TP53, PBRM1, MYT1L, SPTA1, and FAT1) were recurrently mutated between thymic adenocarcinoma and carcinomas. Recurrent mutations in chromatin remodeling genes were reported in thymic carcinomas [45], but no mutation in chromatin remodeling genes (SETD2, KDM6A, MLL2, and MLL3) was found in thymic adenocarcinoma. In addition, we compared SCNAs between thymic adenocarcinoma and 35 thymic carcinomas previously analyzed by array CGH [37, 38, 46]. Five amplifications (chr5p, chr8q, chr12p, chr20p, and chr20q) and six deletions (chr3p, chr6q, chr9p, chr16q, and chr17p) were recurrent (>15%) in thymic carcinomas and adenocarcinoma (Fig. 6b). The focal MYC amplification was remarkable in thymic adenocarcinoma while thymic carcinomas showed broad chr8q amplifications. Chr6p and chr6q deletions, which have been recurrent in TETs [21, 47], were frequent (26 and 37% each) in thymic carcinomas. Instead of an arm-level deletion of chr6p, the focal deletion of HLA class II alleles with the slight amplification of chr6p was found in thymic adenocarcinoma, which was the same genotype with the other case of thymic adenocarcinoma [2].

## Discussion

This is the first sequencing approach discovering the high incidence of cancer-related mutations, which were likely to be associated with aggressive behaviors of thymic adenocarcinoma. First, we found critical driver mutations of TP53 and MYC. TP53 had the p.C176F mutation, which showed remarkable allele frequencies from both WES and WTS, indicating that the TP53 mutant was dominant in the tumor cell population. TP53 mutations have been recurrent in thymic carcinomas and associated with poor survival [44]. The other driver gene, MYC, showed the dramatic focal amplification up to about eight copies with the complex rearrangement of chromosome 8. With the tandem duplication of chr8q, copy number increases seemed to be further accumulated in the end of chr8q, which may be caused by chromosomal instability of tandemly-duplicated chr8q. Considering that such a focal MYC amplification was unprecedented in type A and AB thymomas clinically benign [37, 38, 46], the MYC amplification may contribute to high malignancy of thymic adenocarcinoma.

In addition, we identified the p.R211C mutation of TGFB2, a member of the TGFB family, which performs crucial roles in bone remodeling, even associated with formation of the tumor microenvironment in the bone [48]. The propeptide domain of TGFB forms the dimer known as the latency-associated proteins (LAPs) [34]. Because LAPs that non-covalently hold active TGFB are responsible for activation of latent TGFB, the mutation in LAPs is highly expected to dysregulate TGFB latency. Especially TGFB1 mutations that have occurred in the region close to disulfide bonds (C223 and C225) joining LAPs have been related to dysregulation of active TGFB1. Previously, mutations in the propeptide of TGFB1 (R218C, C223R, and C225R) were reported to induce overexpression of active TGFB1, eventually promoting bone remodeling [35, 36]. Because the current case showed the systemic bone metastasis, we further investigated the p.R211C mutation which is located in the propeptide domain of TGFB2 (Additional file 6: Figure S3). While structures and functions of TGFB1 were extensively studied, TGFB2 was less explored. Hence, we performed the local alignment of TGFB sequences (Additional file 6: Figure S3), to investigate the sequence similarity between the p.R211C mutation of TGFB2 and the previous mutations (R218C, C223R, and C225R) of TGFB1. The p.R211C mutation of TGFB2 occurred in the conserved arginine region close to the TGFB1 mutation sites while the propeptides had some differences in sequence. The p.R211C mutation of the TGFB2 propeptide may dysregulate active TGFB2 in a similar way with the TGFB1 mutations and may be a possible factor for the systemic bone metastasis, requiring further investigation when more samples from thymic adenocarcinoma are available.

With its aggressive characteristics of thymic adenocarcinoma, it showed genetic aberrations remarkably enriched in the immune system including the deletion of HLA class II alleles. Dysfunction of the immune system has been associated with TETs, especially accompanied by autoimmune diseases such as myasthenia gravis (MG) [49]. Loss of HLA class II alleles, expressions of which on thymic epithelial cells are crucial for negative selection of autoreactive T cells, has been considered as a main pathogenic factor of paraneoplastic autoimmunity [47]. Although no case of thymic adenocarcinoma has been accompanied by autoimmune diseases, deletions of HLA class II alleles have been also observed in thymic adenocarcinoma including the current case [2]. Hypothetically, loss of HLA class II alleles may be not only associated with autoimmune characteristics but also likely related to tumorigenesis in the thymus. The reasonable interpretation for the dysregulated immune system in tumor is the tumor immune escape process, which is frequently found in lung cancer [50]. Downregulation of HLA class I molecules has been related to lung cancer in terms of tumor escape from immune surveillance [50]. In the thymus that is a primary lymphoid organ, tumor immune escape mechanisms derived by genetic aberrations may be critical for tumor progression.

## Conclusion

Our first sequence analysis revealed a high incidence of genetic aberrations in cancer-related genes, explaining aggressive characteristics of thymic adenocarcinoma. Mutations in TP53, TGFB2, TNFSF15, MYC, and HLA class II genes were pinpointed. Genetic aberrations affecting the immune system, including deletion of HLA class II genes which was found to be recurrent in thymic adenocarcinoma, were further discussed.

## Additional files

Additional file 1: Table S1. Genomic positions and designed primers for Sanger sequencing validation. (DOCX 14 kb)

Additional file 2: Table S2. Fifty-five somatic mutations detected by WES. (DOCX 19 kb)

Additional file 3: The 6-base pair insertion and somatic SNV in the FAT1 gene. IGV (http://software.broadinstitute.org/software/igv/) shows the distribution of reads at the chromosomal region (chr4:187,527,221–187,527,332), which encompasses exon 17 of FAT1. The 6-base pair insertion (+GACATC) and SNV (G > A) are indicated by the purple ‘I’ and green ‘A’ symbol each. They exist on the same reads, which suggests that the mutations occur at the same allele of FAT1. (PNG 23 kb)

Additional file 4: Figure S2. Sanger sequencing validation for the mutated six genes. Chromatograms of forward (top) and reverse (bottom) sequences for the normal (left) and tumor (right) sample. **(**A) 5 SNVs (TGFB2, TP53, TNFSF15, PEG10, and RNASEL) are validated by Sanger sequencing. Base substitutions and allele frequencies from WES are simultaneously represented above results of the validation. Signal intensities of variants detected by Sanger sequencing are consistent with the results of WES. (B) Sanger sequencing confirms the 6-base pair insertion and one-base substitution of FAT1. Detected base shifts derived from the 6-base pair insertion are indicated along the signal. (PPTX 321 kb)

Additional file 5: Table S3. Occurrences of the TP53 p.C176F variant in the COSMIC database (version 68). (DOCX 12 kb)

Additional file 6: Figure S3. TGFB2 mutation compared to TGFB1 mutations and the latent TGFB structure. (A) The p.R211C mutation of TGFB2 is located in the propeptide domain (red). Three mutations (R218C, C223R, and C225R) of TGFB1 (green) have been reported to affect the TGFB1 latency. (B) The local pairwise alignment between TGFB2 and TGFB1 sequences. The active TGFB1 and TGFB2 domain have similar sequences while their propeptide domain sequences are different. The R211 region is conserved between TGFB1 and TGFB2. (C) The dimer of TGFB forms the latent TGFB structure which regulates the active TGFB. LAP and TGFB are separated proteolytically and LAP regulates the liberation of TGFB noncovalently. The active TGFB liberated from the latent complex is associated with downregulation of immune surveillance and enhancement of tumor invasion and bone remodeling in the malignant tumor. LAP; latency-associated protein, LLC; large latent complex, LTBP; latent TGFB binding protein. (TIFF 388 kb)

Additional file 7: Table S4. Structural variations detected by FACTERA in the tumor and normal sample. (DOCX 15 kb)

Additional file 8: Figure S4. Read distributions at each breakpoint of the structural variation between MCM4 and SNTB1. Near exon 8 of MCM4 (chr8:48,877,987–48,879,365), clipped reads support the somatic fusion event between MCM4 and SNTB1 with about 100X coverage. In intron 1 of SNTB1 (chr8:121,815,027–121,815,715), new reads supporting the somatic fusion event are discovered in the tumor sample with about 75X coverage while the intronic region is not covered by WES in the normal sample. (PPTX 171 kb)

Additional file 9: Table S5. Five focal SCNAs detected by Varscan2-CBS and EXCAVATOR (DOCX 14 kb)

Additional file 10: Figure S5. Two focal SCNAs encompassing less than 10 exons. (A) Focal amplification of the chromosomal region (chr19:9,045,965–9,089,201) detected by Varscan2-CBS. Copy number bins at the region show the significantly-amplified copy number state from the neutral state. The region includes exon 1 to exon 5 of the MUC16 gene. (B) Focal amplification of the chromosomal region (chrX:135,426,776–135,432,395). The amplified region (5 kb) is supported by 26 copy number bins and encompasses exon 6 of the GPR112 gene. (PPTX 239 kb)

Additional file 11: Table S6. Two fusion transcripts detected by deFuse (DOCX 13 kb)

Additional file 12: Figure S6. The CTBS-GNG5 fusion transcript by intergenic splicing. The GNG5 gene (blue) and CTBS gene (red) are consecutively located in chromosome 6 via the intergenic region (64 kb). The read-through transcript is transcribed from their DNA sequences without termination between CTBS and GNG5. The region from the tail of CTBS exon 6 to the head of GNG5 exon 3 is spliced out across the intergenic region by the intergenic splicing event. The transcript possesses a fusion sequence between CTBS exon 6 and GNG5 exon 3. The CTBS-GNG5 fusion sequence is supported by 76 reads with a uniform read distribution (PDF 57 kb)

Additional file 13: Table S7. The set of total 39 genes for enrichment analysis (DOCX 15 kb)

Additional file 14: Table S8. Fifty-two GO terms enriched in the biological process (DOCX 20 kb)

Additional file 15: Table S9. Enriched KEGG and Reactome pathways (DOCX 14 kb)

## Abbreviations

HLA: Human leukocyte antigen
TETs: Thymic epithelial tumors
Array CGH: Array comparative genomic hybridization
WES: Whole exome sequencing
WTS: Whole transcriptome sequencing
SNVs: Somatic single nucleotide variants
SNPs: Single nucleotide polymorphisms
CBS: Circular binary segmentation
SCNAs: Somatic copy number alterations
PET/CT: Positron emission tomography/computed tomography
IHC: Immunohistochemistry
H&E: Hematoxylin and eosin
IGV: Integrative Genomics Viewer
LAPs: Latency-associated proteins
MG: Myasthenia gravis
