# Supplementary material for: Characterization of genetic aberrations in a single case of metastatic thymic adenocarcinoma
Source: BMC Cancer. 2017 May 15;17:330. doi: 10.1186/s12885-017-3282-9 (PMC5432996; doi:10.1186/s12885-017-3282-9)
Supplement: Supplementary file 1 — Genomic positions and designed primers for Sanger sequencing validation. (DOCX 14 kb) [file 12885_2017_3282_MOESM1_ESM.docx]

**Table S1. Genomic positions and designed primers for Sanger sequencing validation**

| **Index** | **Primer_name**  **F:forward R:reverse** | **Genomic position** | **Sequence** | **PCR_size (bp)** | **Annealing**  **temperature** |
| --- | --- | --- | --- | --- | --- |
| **1** | **TGFB2_F** | chr1:218607460-218607461 | AAATTTAGGTAATGAATTAGAACACTG | 260 | 60 |
|  | **TGFB2_R** |  | GGGAGGAAAAGAGAGAGTGG |  |  |
| **2** | **TP53_F** | chr17:7578403-7578404 | GCCAGACCTAAGAGCAATCA | 265 | 60 |
|  | **TP53_R** |  | CTGCCCTCAACAAGATGTTT |  |  |
| **3** | **TNFSF15_F** | chr9:117552937-117552938 | ACCATTAGCTTGTCCCCTTC | 263 | 60 |
|  | **TNFSF15_R** |  | ATCCCAGAGTCGGGAGACTA |  |  |
| **4** | **PEG10_F** | chr7:94293260-94293261 | AGACATGCTGGCTCCTTTC | 244 | 60 |
|  | **PEG10_R** |  | GATCTTGCGTTTGGCAAC |  |  |
| **5** | **RNASEL_F** | chr1:182554694-182554695 | CCTCATTTTCCACATCTTCC | 249 | 60 |
|  | **RNASEL_R** |  | CTTCAGAAGGAGGCATCTACC |  |  |
| **6** | **FAT1_F** | chr4:187527274-187527275 | GAAAAGCAACAGAGGCCAAT | 247 | 60 |
|  | **FAT1_R** |  | TGAACGTGCATTCTGTCTTT |  |  |
| **7** | **MCM4-SNTB1_F** | fusion sequence  chr8:121816210-121816370  chr8:48878682-48878801 | TCCTCTTTCGTCAACCATCT | 279 | 60 |
|  | **MCM4-SNTB1_R** |  | ATCAGCTGGGATGTCCTGAT |  |  |
