# Supplementary material for: Characterization of genetic aberrations in a single case of metastatic thymic adenocarcinoma
Source: BMC Cancer. 2017 May 15;17:330. doi: 10.1186/s12885-017-3282-9 (PMC5432996; doi:10.1186/s12885-017-3282-9)
Supplement: Supplementary file 2 — Fifty-five somatic mutations detected by WES. (DOCX 19 kb) [file 12885_2017_3282_MOESM2_ESM.docx]

**Table S2. Fifty-five somatic mutations detected by WES**

| **Index** | **Mutation type** | **Coding change** | **Chr** | **Start position** | **End position** | **Reference sequence** | **Variant sequence** |
| --- | --- | --- | --- | --- | --- | --- | --- |
| 1 | nonsynonymous SNV | EXTL1:NM_004455:exon1:c.C308T:p.A103V | chr1 | 26349445 | 26349445 | C | T |
| 2 | nonsynonymous SNV | A3GALT2:NM_001080438:exon5:c.C796T:p.R266C | chr1 | 33772594 | 33772594 | G | A |
| 3 | nonsynonymous SNV | APH1A:NM_001077628:exon4:c.T425A:p.L142H,APH1A:NM_016022:exon4:c.T425A:p.L142H APH1A:NM_001243771:exon3:c.T254A:p.L85H,APH1A:NM_001243772:exon3:c.T215A:p.L72H | chr1 | 150239812 | 150239812 | A | T |
| 4 | synonymous SNV | OR10T2:NM_001004475:exon1:c.G63T:p.L21L | chr1 | 158369194 | 158369194 | C | A |
| 5 | nonsynonymous SNV | SPTA1:NM_003126:exon19:c.C2759G:p.P920R | chr1 | 158627313 | 158627313 | G | C |
| 6 | nonsynonymous SNV | RNASEL:NM_021133:exon2:c.C1248G:p.H416Q | chr1 | 182554694 | 182554694 | G | C |
| 7 | synonymous SNV | REN:NM_000537:exon10:c.C1206T:p.F402F | chr1 | 204124159 | 204124159 | G | A |
| 8 | nonsynonymous SNV | TGFB2:NM_003238:exon3:c.C547T:p.R183C,TGFB2:NM_001135599:exon4:c.C631T:p.R211C | chr1 | 218607460 | 218607460 | C | T |
| 9 | unknown | UNKNOWN | chr1 | 223815812 | 223815812 | G | T |
| 10 | synonymous SNV | MYT1L:NM_015025:exon9:c.C429T:p.D143D | chr2 | 1946830 | 1946830 | G | A |
| 11 | synonymous SNV | DPP4:NM_001935:exon18:c.C1510T:p.L504L | chr2 | 162873335 | 162873335 | G | A |
| 12 | synonymous SNV | ITPR1:NM_001099952:exon8:c.C558G:p.P186P,ITPR1:NM_001168272:exon8:c.C558G:p.P186P ITPR1:NM_002222:exon8:c.C558G:p.P186P | chr3 | 4685852 | 4685852 | C | G |
| 13 | synonymous SNV | PBRM1:NM_018313:exon15:c.G1776A:p.L592L | chr3 | 52651320 | 52651320 | C | T |
| 14 | nonsynonymous SNV | OR5H2:NM_001005482:exon1:c.C323A:p.S108Y | chr3 | 98002054 | 98002054 | C | A |
| 15 | nonsynonymous SNV | TENM3:NM_001080477:exon27:c.C7855T:p.R2619C | chr4 | 183721259 | 183721259 | C | T |
| 16 | synonymous SNV | PCDHAC1:NM_018898:exon1:c.G1659A:p.P553P,PCDHAC1:NM_031882:exon1:c.G1659A:p.P553P | chr5 | 140308136 | 140308136 | G | A |
| 17 | synonymous SNV | UTRN:NM_007124:exon70:c.G9894A:p.P3298P | chr6 | 145157506 | 145157506 | G | A |
| 18 | nonsynonymous SNV | PEG10:NM_001184962:exon2:c.G494A:p.R165H,PEG10:NM_001040152:exon2:c.G392A:p.R131H PEG10:NM_001172438:exon2:c.G620A:p.R207H | chr7 | 94293260 | 94293260 | G | A |
| 19 | synonymous SNV | CPA4:NM_001163446:exon10:c.C1002T:p.I334I,CPA4:NM_016352:exon11:c.C1101T:p.I367I | chr7 | 129962351 | 129962351 | C | T |
| 20 | nonsynonymous SNV | GPR124:NM_032777:exon14:c.T2092A:p.S698T | chr8 | 37695290 | 37695290 | T | A |
| 21 | nonsynonymous SNV | MAFA:NM_201589:exon1:c.C401T:p.T134M | chr8 | 144512176 | 144512176 | G | A |
| 22 | nonsynonymous SNV | NOL6:NM_022917:exon21:c.A2776T:p.T926S | chr9 | 33464880 | 33464880 | T | A |
| 23 | nonsynonymous SNV | TNFSF15:NM_001204344:exon2:c.T374C:p.V125A,TNFSF15:NM_005118:exon4:c.T551C:p.V184A | chr9 | 117552937 | 117552937 | A | G |
| 24 | nonsynonymous SNV | ZBTB34:NM_001099270:exon2:c.G1300A:p.G434R | chr9 | 129642990 | 129642990 | G | A |
| 25 | nonsynonymous SNV | OR2AG1:NM_001004489:exon1:c.C259T:p.R87C | chr11 | 6806527 | 6806527 | C | T |
| 26 | synonymous SNV | NADSYN1:NM_018161:exon14:c.G1281A:p.T427T | chr11 | 71194025 | 71194025 | G | A |
| 27 | synonymous SNV | KCNC2:NM_001260497:exon2:c.C543T:p.D181D,KCNC2:NM_001260499:exon2:c.C543T:p.D181D KCNC2:NM_153748:exon2:c.C543T:p.D181D,KCNC2:NM_139137:exon2:c.C543T:p.D181D KCNC2:NM_139136:exon2:c.C543T:p.D181D,KCNC2:NM_001260498:exon2:c.C543T:p.D181D | chr12 | 75601221 | 75601221 | G | A |
| 28 | synonymous SNV | FOXG1:NM_005249:exon1:c.T1365A:p.S455S | chr14 | 29237850 | 29237850 | T | A |
| 29 | synonymous SNV | GABRB3:NM_001278631:exon9:c.C732T:p.Y244Y,GABRB3:NM_021912:exon8:c.C987T:p.Y329Y GABRB3:NM_000814:exon8:c.C987T:p.Y329Y,GABRB3:NM_001191320:exon6:c.C732T:p.Y244Y GABRB3:NM_001191321:exon6:c.C774T:p.Y258Y | chr15 | 26806172 | 26806172 | G | A |
| 30 | nonsynonymous SNV | ONECUT1:NM_004498:exon1:c.C82A:p.L28M | chr15 | 53082000 | 53082000 | G | T |
| 31 | nonsynonymous SNV | CASKIN1:NM_020764:exon19:c.T3892A:p.S1298T | chr16 | 2229210 | 2229210 | A | T |
| 32 | synonymous SNV | RBFOX1:NM_001142334:exon4:c.A400C:p.R134R,RBFOX1:NM_145891:exon3:c.A460C:p.R154R RBFOX1:NM_001142333:exon6:c.A400C:p.R134R,RBFOX1:NM_018723:exon6:c.A400C:p.R134R RBFOX1:NM_145892:exon3:c.A460C:p.R154R,RBFOX1:NM_145893:exon3:c.A460C:p.R154R | chr16 | 7629908 | 7629908 | A | C |
| 33 | nonsynonymous SNV | ZNF843:NM_001136509:exon2:c.G340A:p.E114K | chr16 | 31447831 | 31447831 | C | T |
| 34 | synonymous SNV | IRX3:NM_024336:exon2:c.G822A:p.A274A | chr16 | 54318971 | 54318971 | C | T |
| 35 | nonsynonymous SNV | ANKFY1:NM_016376:exon12:c.C1613T:p.A538V,ANKFY1:NM_001257999:exon12:c.C1739T:p.A580V | chr17 | 4088199 | 4088199 | G | A |
| 36 | nonsynonymous SNV | TP53:NM_001276699:exon1:c.G50T:p.C17F,TP53:NM_001126118:exon4:c.G410T:p.C137F TP53:NM_001126112:exon5:c.G527T:p.C176F,TP53:NM_001276761:exon5:c.G410T:p.C137F TP53:NM_001126115:exon1:c.G131T:p.C44F,TP53:NM_001126113:exon5:c.G527T:p.C176F TP53:NM_001276697:exon1:c.G50T:p.C17F,TP53:NM_001276695:exon5:c.G410T:p.C137F TP53:NM_001276760:exon5:c.G410T:p.C137F,TP53:NM_001126117:exon1:c.G131T:p.C44F TP53:NM_001126114:exon5:c.G527T:p.C176F,TP53:NM_000546:exon5:c.G527T:p.C176F TP53:NM_001276696:exon5:c.G410T:p.C137F,TP53:NM_001276698:exon1:c.G50T:p.C17F  TP53:NM_001126116:exon1:c.G131T:p.C44F | chr17 | 7578403 | 7578403 | C | A |
| 37 | synonymous SNV | DNAH17:NM_173628:exon54:c.C8334T:p.D2778D | chr17 | 76471537 | 76471537 | G | A |
| 38 | synonymous SNV | RNF213:NM_001256071:exon26:c.G5295A:p.P1765P | chr17 | 78313462 | 78313462 | G | A |
| 39 | synonymous SNV | PNPLA6:NM_001166112:exon17:c.C1710T:p.A570A,PNPLA6:NM_001166111:exon17:c.C1932T:p.A644A PNPLA6:NM_001166114:exon15:c.C1905T:p.A635A,PNPLA6:NM_001166113:exon18:c.C1788T:p.A596A  PNPLA6:NM_006702:exon18:c.C1788T:p.A596A | chr19 | 7615274 | 7615274 | C | T |
| 40 | synonymous SNV | NWD1:NM_001007525:exon8:c.C2016T:p.S672S,NWD1:NM_001290355:exon9:c.C1611T:p.S537S | chr19 | 16872832 | 16872832 | C | T |
| 41 | nonsynonymous SNV | WDR87:NM_001291088:exon4:c.C2005G:p.L669V,WDR87:NM_031951:exon4:c.C1888G:p.L630V | chr19 | 38384338 | 38384338 | G | C |
| 42 | synonymous SNV | MARK4:NM_001199867:exon11:c.C1074T:p.N358N,MARK4:NM_031417:exon11:c.C1074T:p.N358N | chr19 | 45783699 | 45783699 | C | T |
| 43 | synonymous SNV | TSKS:NM_021733:exon5:c.C633T:p.N211N | chr19 | 50250676 | 50250676 | G | A |
| 44 | synonymous SNV | ZSCAN1:NM_182572:exon6:c.C1155T:p.C385C | chr19 | 58565347 | 58565347 | C | T |
| 45 | nonsynonymous SNV | GZF1:NM_022482:exon1:c.C1045T:p.R349C | chr20 | 23346065 | 23346065 | C | T |
| 46 | nonsynonymous SNV | DEFB121:NM_001171832:exon2:c.G139T:p.V47L,DEFB121:NM_001011878:exon2:c.G175T:p.V59L | chr20 | 29992772 | 29992772 | C | A |
| 47 | synonymous SNV | IL17RA:NM_014339:exon3:c.C213T:p.S71S,IL17RA:NM_001289905:exon3:c.C213T:p.S71S | chr22 | 17578736 | 17578736 | C | T |
| 48 | ncRNA_exonic | TPRXL | chr3 | 14106309 | 14106309 | C | T |
| 49 | ncRNA_exonic | TPRXL | chr3 | 14106332 | 14106332 | G | C |
| 50 | ncRNA_exonic | TPRXL | chr3 | 14106354 | 14106354 | C | T |
| 51 | ncRNA_exonic | LINC00967 | chr8 | 67105533 | 67105533 | G | A |
| 52 | ncRNA_exonic | COLCA1 | chr11 | 111166198 | 111166198 | G | A |
| 53 | splicing | SEL1L2(NM_001271539:exon13:c.1104+1G>A) | chr20 | 13856683 | 13856683 | C | T |
| 54 | nonframeshift insertion | FAT1:NM_005245:exon17:c.10299_10300insGATGTC:p.N3434delinsDVN | chr4 | 187527274 | 187527274 | - | GACATC |
| 55 | frameshift insertion | OR5D16:NM_001005496:exon1:c.309_310insG:p.F103fs | chr11 | 55606536 | 55606536 | - | G |
