# Supplementary figures and images for: Characterization of genetic aberrations in a single case of metastatic thymic adenocarcinoma
Source: BMC Cancer. 2017 May 15;17:330. doi: 10.1186/s12885-017-3282-9 (PMC5432996; doi:10.1186/s12885-017-3282-9)

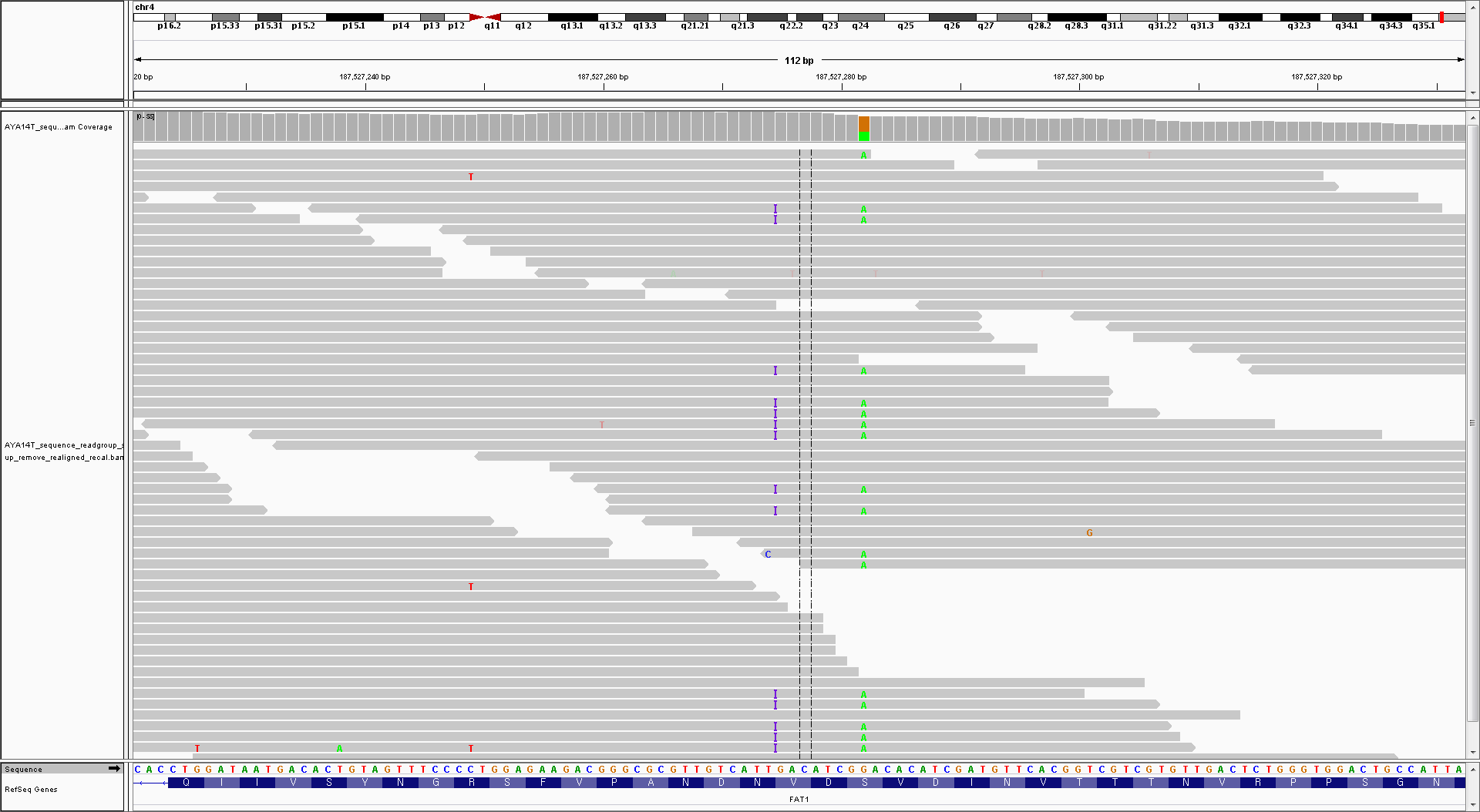

Supplement: Supplementary file 3 — The 6-base pair insertion and somatic SNV in the FAT1 gene. IGV (http://software.broadinstitute.org/software/igv/) shows the distribution of reads at the chromosomal region (chr4:187,527,221–187,527,332), which encompasses exon 17 of FAT1. The 6-base pair insertion (+GACATC) and SNV (G > A) are indicated by the purple ‘I’ and green ‘A’ symbol each. They exist on the same reads, which suggests that the mutations occur at the same allele of FAT1. (PNG 23 kb) [file 12885_2017_3282_MOESM3_ESM.png]

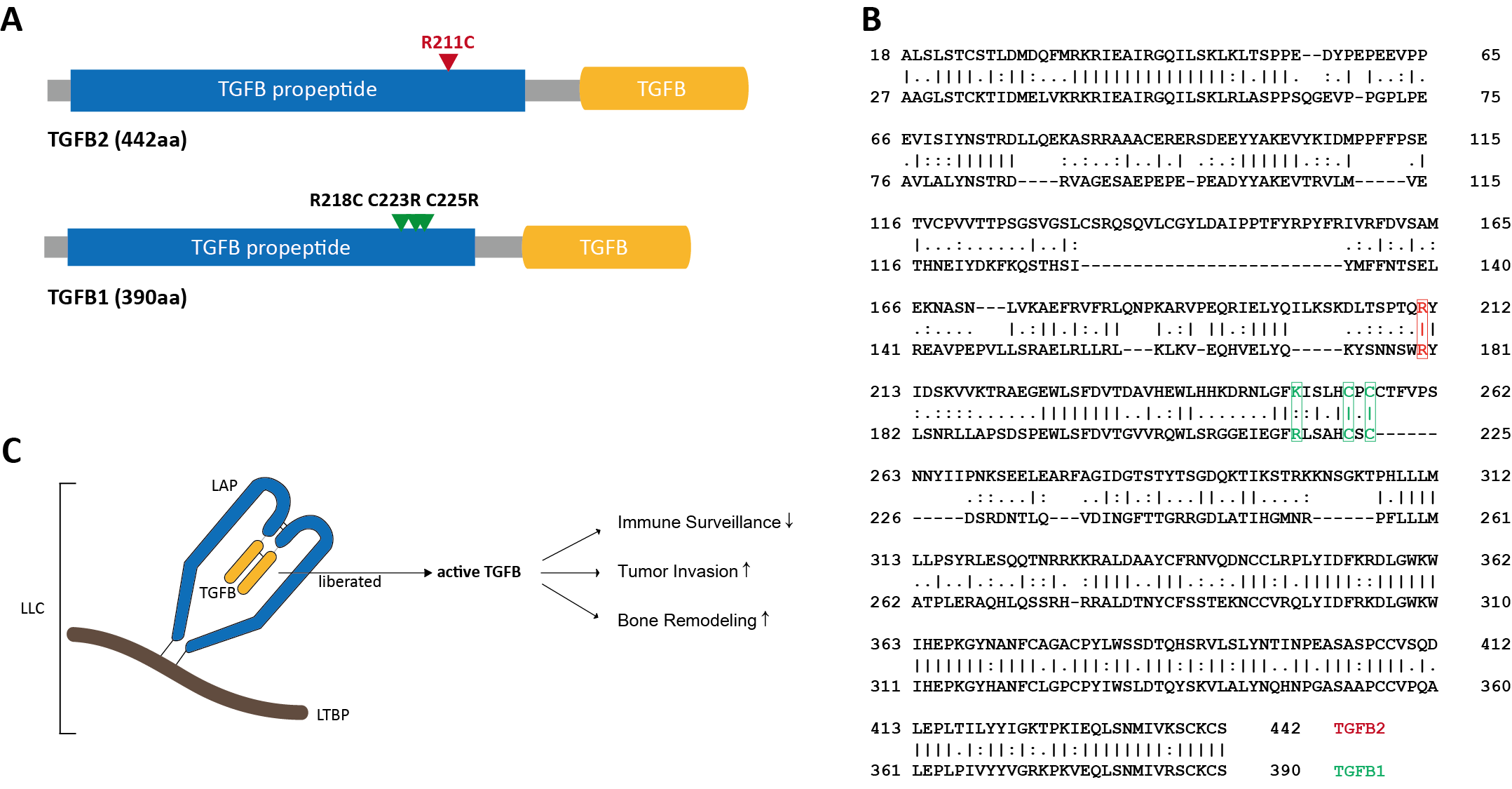

Supplement: Supplementary file 6 — TGFB2 mutation compared to TGFB1 mutations and the latent TGFB structure. (A) The p.R211C mutation of TGFB2 is located in the propeptide domain (red). Three mutations (R218C, C223R, and C225R) of TGFB1 (green) have been reported to affect the TGFB1 latency. (B) The local pairwise alignment between TGFB2 and TGFB1 sequences. The active TGFB1 and TGFB2 domain have similar sequences while their propeptide domain sequences are different. The R211 region is conserved between TGFB1 and TGFB2. (C) The dimer of TGFB forms the latent TGFB structure which regulates the active TGFB. LAP and TGFB are separated proteolytically and LAP regulates the liberation of TGFB noncovalently. The active TGFB liberated from the latent complex is associated with downregulation of immune surveillance and enhancement of tumor invasion and bone remodeling in the malignant tumor. LAP; latency-associated protein, LLC; large latent complex, LTBP; latent TGFB binding protein. (TIFF 388 kb) [file 12885_2017_3282_MOESM6_ESM.tif]

## Slide 1
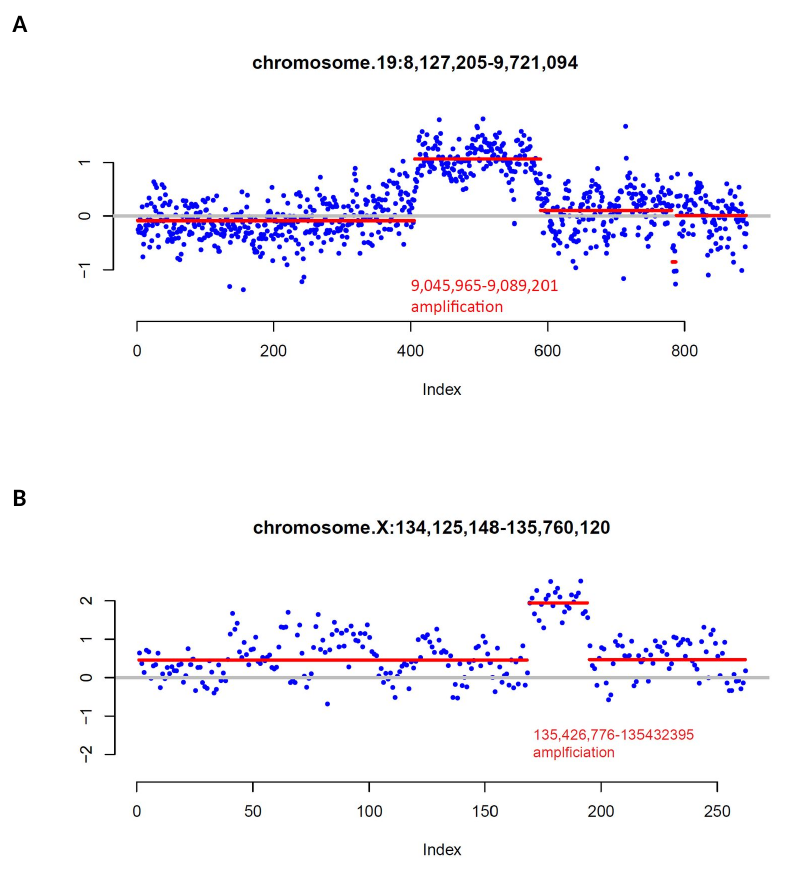

A
B

Supplement: Supplementary file 10 — Two focal SCNAs encompassing less than 10 exons. (A) Focal amplification of the chromosomal region (chr19:9,045,965–9,089,201) detected by Varscan2-CBS. Copy number bins at the region show the significantly-amplified copy number state from the neutral state. The region includes exon 1 to exon 5 of the MUC16 gene. (B) Focal amplification of the chromosomal region (chrX:135,426,776–135,432,395). The amplified region (5 kb) is supported by 26 copy number bins and encompasses exon 6 of the GPR112 gene. (PPTX 239 kb) [file 12885_2017_3282_MOESM10_ESM.pptx]
