# Supplementary material for: Characterization of genetic aberrations in a single case of metastatic thymic adenocarcinoma
Source: BMC Cancer. 2017 May 15;17:330. doi: 10.1186/s12885-017-3282-9 (PMC5432996; doi:10.1186/s12885-017-3282-9)
Supplement: Supplementary file 4 — Sanger sequencing validation for the mutated six genes. Chromatograms of forward (top) and reverse (bottom) sequences for the normal (left) and tumor (right) sample. (A) 5 SNVs (TGFB2, TP53, TNFSF15, PEG10, and RNASEL) are validated by Sanger sequencing. Base substitutions and allele frequencies from WES are simultaneously represented above results of the validation. Signal intensities of variants detected by Sanger sequencing are consistent with the results of WES. (B) Sanger sequencing confirms the 6-base pair insertion and one-base substitution of FAT1. Detected base shifts derived from the 6-base pair insertion are indicated along the signal. (PPTX 321 kb) [file 12885_2017_3282_MOESM4_ESM.pptx]

## Slide 1
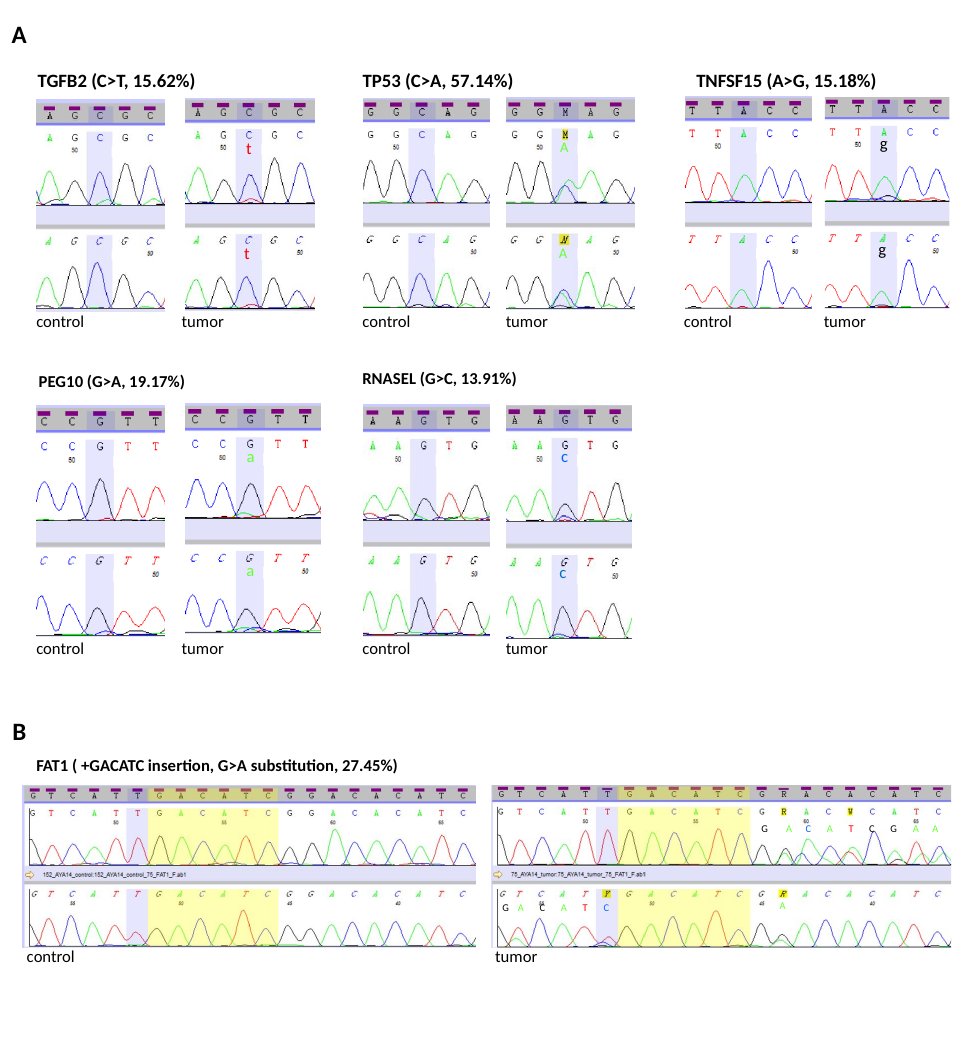

A
TGFB2 (C>T, 15.62%)
TP53 (C>A, 57.14%)
TNFSF15 (A>G, 15.18%)
g
g
A
A
t
t
control
tumor
control
tumor
control
tumor
RNASEL (G>C, 13.91%)
PEG10 (G>A, 19.17%)
a
a
c
c
control
tumor
control
tumor
B
FAT1 ( +GACATC insertion, G>A substitution, 27.45%)
G A C A T C G A A
A
G A C A T C
tumor
control
