# Supplementary material for: Characterization of genetic aberrations in a single case of metastatic thymic adenocarcinoma
Source: BMC Cancer. 2017 May 15;17:330. doi: 10.1186/s12885-017-3282-9 (PMC5432996; doi:10.1186/s12885-017-3282-9)
Supplement: Supplementary file 5 — Occurrences of the TP53 p.C176F variant in the COSMIC database (version 68). (DOCX 12 kb) [file 12885_2017_3282_MOESM5_ESM.docx]

**Table S3. Occurrences of the TP53 p.C176F variant in the COSMIC database (version 68).**

| **COSMIC v68**  **ID** | **COSMIC v68**  **Occurrence** | **Chromosome** | **Position** | **Mutation type** | **refSeq genes** |
| --- | --- | --- | --- | --- | --- |
| COSM117396  COSM117398  COSM1640850  COSM117397  COSM117395  COSM10645 | 6(ovary)  3(bone)  5(breast)  10(stomach)  1(haematopoietic_and_lymphoid_tissue)  7(urinary_tract)  2(pancreas)  5(liver)  2(genital_tract)  20(oesophagus)  1(adrenal_gland)  1(skin)  2(prostate)  15(lung)  21(upper_aerodigestive_tract)  18(large_intestine)  3(central_nervous_system) | chr17 | 7578403 | nonsynonymous | TP53:NM_001276699:exon1:c.G50T:p.C17F  TP53:NM_001126118:exon4:c.G410T:p.C137F  TP53:NM_001126112:exon5:c.G527T:p.C176F  TP53:NM_001276761:exon5:c.G410T:p.C137F  TP53:NM_001126115:exon1:c.G131T:p.C44F  TP53:NM_001126113:exon5:c.G527T:p.C176F  TP53:NM_001276697:exon1:c.G50T:p.C17F  TP53:NM_001276695:exon5:c.G410T:p.C137F  TP53:NM_001276760:exon5:c.G410T:p.C137F  TP53:NM_001126117:exon1:c.G131T:p.C44F  TP53:NM_001126114:exon5:c.G527T:p.C176F  TP53:NM_000546:exon5:c.G527T:p.C176F  TP53:NM_001276696:exon5:c.G410T:p.C137F  TP53:NM_001276698:exon1:c.G50T:p.C17F  TP53:NM_001126116:exon1:c.G131T:p.C44F |
