# Supplementary material for: Characterization of genetic aberrations in a single case of metastatic thymic adenocarcinoma
Source: BMC Cancer. 2017 May 15;17:330. doi: 10.1186/s12885-017-3282-9 (PMC5432996; doi:10.1186/s12885-017-3282-9)
Supplement: Supplementary file 7 — Structural variations detected by FACTERA in the tumor and normal sample. (DOCX 15 kb) [file 12885_2017_3282_MOESM7_ESM.docx]

**Table S4. Structural variations detected by FACTERA in the tumor and normal sample.**

| tumor |  |  | |  | |  | |  | |  | |  | |  |  | |  |  |  |
| --- | --- | --- | --- | --- | --- | --- | --- | --- | --- | --- | --- | --- | --- | --- | --- | --- | --- | --- | --- |
| Est_Type | **Region1** | | **Region2** | | **Break1** | | **Break2** | | **Break**  **support1** | | **Break**  **support2** | | **Orientation** | | | **Fusion_seq** | | | |
| - | SNTB1 | | MCM4 | | chr8:121816370 | | chr8:48878682 | | 40 | | 41 | | 1+ 2+ | | | GTCTAATGTTTACCACCACAGTCCAGTAG  TATGAGTGGTGGATGATGTGA | | | |
| DEL | TRBV7-7 | | TRBV7-3 | | chr7:142119948 | | chr7:142247245 | | 16 | | 15 | | 1+ 2+ | | | CTCTGCAGAGAACCGATCACTGGGCAGCC  CTGATTTGTCTGGTGCACTGT | | | |
| DEL | CLCNKB | | FAM131C | | chr1:16376516 | | chr1:16386258 | | 25 | | 10 | | 1+ 2+ | | | TCCCAACCTTATGTAGAAAGCTCTACCCG  CCACCTGAGCCCCTAAAGCCC | | | |
|  |  | |  | |  | |  | |  | |  | |  | | |  | | | |
|  |  | |  | |  | |  | |  | |  | |  | | |  | | | |
|  |  | |  | |  | |  | |  | |  | |  | | |  | | | |
| normal |  | |  | |  | |  | |  | |  | |  | | |  | | | |
| Est_Type | **Region1** | | **Region2** | | **Break1** | | **Break2** | | **Break**  **support1** | | **Break**  **support2** | | **Orientation** | | | **Fusion_seq** | | | |
| TRA | IPO11 | | TRANK1 | | chr5:61857118 | | chr3:37007929 | | 46 | | 10 | | 1+ 2+ | | | TGCCATCTGATAATAGGTGAGGAAATGTTT  TCTTAAAATTTGTTTCTTTC | | | |
| DEL | TRBV7-7 | | TRBV7-3 | | chr7:142119948 | | chr7:142247245 | | 17 | | 20 | | 1+ 2+ | | | CTCTGCAGAGAACCGATCACTGGGCAGCCCT  GATTTGTCTGGTGCACTGT | | | |
| DEL | TRBV7-6 | | TRBV7-3 | | chr7:142139403 | | chr7:142247245 | | 13 | | 20 | | 1+ 2+ | | | CCTCAGGCCTCTCTGCAGAGAACCGATCATT  GGGCAGCCCTGATTTGTCT | | | |
| DEL | CLCNKB | | FAM131C | | chr1:16376516 | | chr1:16386258 | | 21 | | 13 | | 1+ 2+ | | | TCCCAACCTTATGTAGAAAGCTCTACCCGCC  ACCTGAGCCCCTAAAGCCC | | | |
