# Supplementary material for: Characterization of genetic aberrations in a single case of metastatic thymic adenocarcinoma
Source: BMC Cancer. 2017 May 15;17:330. doi: 10.1186/s12885-017-3282-9 (PMC5432996; doi:10.1186/s12885-017-3282-9)
Supplement: Supplementary file 8 — Read distributions at each breakpoint of the structural variation between MCM4 and SNTB1. Near exon 8 of MCM4 (chr8:48,877,987–48,879,365), clipped reads support the somatic fusion event between MCM4 and SNTB1 with about 100X coverage. In intron 1 of SNTB1 (chr8:121,815,027–121,815,715), new reads supporting the somatic fusion event are discovered in the tumor sample with about 75X coverage while the intronic region is not covered by WES in the normal sample. (PPTX 171 kb) [file 12885_2017_3282_MOESM8_ESM.pptx]

## Slide 1
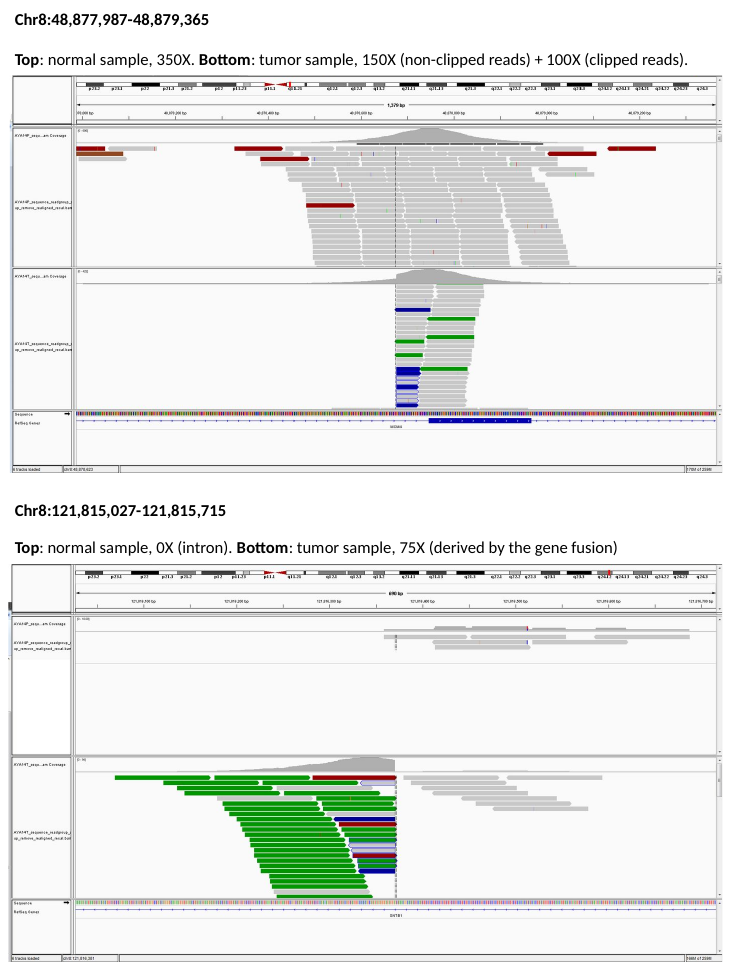

Chr8:48,877,987-48,879,365
Top: normal sample, 350X. Bottom: tumor sample, 150X (non-clipped reads) + 100X (clipped reads).
Chr8:121,815,027-121,815,715
Top: normal sample, 0X (intron). Bottom: tumor sample, 75X (derived by the gene fusion)
