# Supplementary material for: Characterization of genetic aberrations in a single case of metastatic thymic adenocarcinoma
Source: BMC Cancer. 2017 May 15;17:330. doi: 10.1186/s12885-017-3282-9 (PMC5432996; doi:10.1186/s12885-017-3282-9)
Supplement: Supplementary file 9 — Five focal SCNAs detected by Varscan2-CBS and EXCAVATOR (DOCX 14 kb) [file 12885_2017_3282_MOESM9_ESM.docx]

| **Annotated gene*** | | **Varscan2-CBS** | | | | | | | **EXCAVATOR** | | | |
| --- | --- | --- | --- | --- | --- | --- | --- | --- | --- | --- | --- | --- |
| **gene** | **COSMIC gene** | **Chr** | **Start** | **End** | **Size** | **Bins** | **UTR3 bins** | **Seg**  **mean** | **Chr** | **Start** | **End** | **Seg**  **mean** |
| ADCY8,ASAP1,EFR3A,  FAM49B,FAM84B,GSDMC,  HHLA1,KCNQ3,KHDRBS3,  KIAA0196,LRRC6,MTSS1,  MYC,NDRG1,NDUFB9,  NSMCE2,OC90,PHF20L1,  POU5F1B,RNF139,SLA,  SQLE,ST3GAL1,TATDN1,  TG,TMEM65,TMEM71,  TRIB1,TRMT12,WISP1,  ZFAT,ZNF572 | MYC NDRG1 | chr8q  24.21-  24.22 | 125342934 | 136659704 | 11.3Mb | 896 | 23.13% | 1.8881 | chr8q  24.2 | 124780791 | 144826024 | 1.2809 |
| ARC,BAI1,C8orf31,  CCDC166,CYP11B1,  CYP11B2,EEF1D,FAM83H,  GLI4,GML,GPIHBP1,GPR20,  GSDMD,JRK,LY6D,LY6E,  LY6H,LY6K,LYNX1,  LYPD2,MAFA,MAPK15,  MROH5,MROH6,NAPRT,  PSCA,PTP4A3,PYCRL,  RHPN1,SLC45A4,SLURP1,  THEM6,TIGD5,TOP1MT,  TSNARE1,TSTA3,ZC3H3,  ZFP41,ZNF623,ZNF696,  ZNF707 |  | chr8q  24.3 | 142222166 | 144821849 | 2.6Mb | 799 | 22.56% | 1.1476 | chr8q  24.2 | 124780791 | 144826024 | 1.2809 |
| BTNL2,C6orf10,GPSM3,HLA-DOB,HLA-DQA1,HLA-DQA2, HLA-DQB1,HLA-DQB2,HLA-DRA,HLA-DRB1,HLA-DRB5,  NOTCH4,PBX2,TAP2 |  | chr6p  21.32 | 32153267 | 32793889 | 0.6Mb | 29 | 24.13% | -1.2664 | chr6p  21.32 | 32605061 | 32713262 | -0.8458 |
| MUC16 |  | chr19p  13.2 | 9045965 | 9089201 | 0.04Mb | 184 | 0% | 1.0662 | chr19p  13.2-12 | 281501 | 24309566 | -0.0605 |
| GPR112 |  | chrXq  26.3 | 135426776 | 135432395 | 0.005Mb | 26 | 0% | 1.9428 | chrXq  26.3-2 | 114883710 | 154754030 | 0.3843 |

**Table S5. Five focal SCNAs detected by Varscan2-CBS and EXCAVATOR**

*All the genes in the copy number boundaries from Varscan2-CBS were annotated.
