# Supplementary material for: Characterization of genetic aberrations in a single case of metastatic thymic adenocarcinoma
Source: BMC Cancer. 2017 May 15;17:330. doi: 10.1186/s12885-017-3282-9 (PMC5432996; doi:10.1186/s12885-017-3282-9)
Supplement: Supplementary file 11 — Two fusion transcripts detected by deFuse (DOCX 13 kb) [file 12885_2017_3282_MOESM11_ESM.docx]

**Table S6. Two fusion transcripts detected by deFuse**

| **Splitr**  **sequence** | **Splitr**  **count** | **Gene**  **location1** | **Gene**  **location2** | **Gene**  **name1** | **Gene**  **name2** | **Genomic**  **break1** | **Genomic**  **break2** | **Strand1** | **Strand2** | **Read-**  **through** | **Span**  **count** | **probability** |
| --- | --- | --- | --- | --- | --- | --- | --- | --- | --- | --- | --- | --- |
| AAAGAATCAAGCACTTTTCGAAACATTGAAGTTGTTTTTGAACTTGGTGTCACCTTTAATTACAATCTAGCAGACGGAACTGAACTCAGGGGGACCTGGAGCCTTGAGGGAAATAAACTTATTGGAAAATTCAAACGGACAGACAATGGAAACGAACTGAATACTGTCCGAGAAATTATAGGTGATGAACTAGTCCAGACTT\|GTTCGCTGATATCTAAATTAAGAAGTTGGTTCTTGAGTGAATTCTGAAAATGGCTACAAACTTCTTGAATAAAGAAGACAGGACTCTCAATAGAAGAATTTCACATCTCCAAGGGACCCTTCCTTTCATTTTACACTTTGTTACTAATTTGCAGAACTCTATTAATTGGGTAGGATTTCACCC | 55 | Coding | Utr3p | FABP2 | C4orf3 | 120240691 | 120219934 | - | + | Y | 22 | 0.990604 |
| ATACCTGCCTGAATCTGTCTGAGGATCATGTTTGTACCATTGCAAAAGTCCCTTTCCGGGGGGCTCCTTGTAGTGACGCTGCAGGACGTCAGGTGCCCTACAAAACGATCATGAAGCAAATAAATAGTTCTATTTCTGGAAACCTATGGGATAAAGATCAGCGGGCTCCTTATTATAACTATAAAG\|TTTCCCAGGCAGCTGCAGACTTGAAACAGTTCTGTCTGCAGAATGCTCAACATGACCCTCTGCTGACTGGAGTATCTTCAAGTACAAATCCCTTCAGACCCCAGAAAGTCTGTTCCTTTTTGTAGTAAAATGAATCTTTCAAAGGTTTCCCAAACCACTCCTTATGATCCAGTGAATATTCAAGAGAGCTACA | 66 | Coding | Coding | CTBS | GNG5 | 85028940 | 84967653 | - | + | Y | 31 | 0.859537 |
