# Supplementary material for: Characterization of genetic aberrations in a single case of metastatic thymic adenocarcinoma
Source: BMC Cancer. 2017 May 15;17:330. doi: 10.1186/s12885-017-3282-9 (PMC5432996; doi:10.1186/s12885-017-3282-9)
Supplement: Supplementary file 13 — The set of total 39 genes for enrichment analysis (DOCX 15 kb) [file 12885_2017_3282_MOESM13_ESM.docx]

**Table S7. The set of total 39 genes for enrichment analysis**

| **UserID** | **Gene Symbol** | **Gene name** | **Mutation Type** |
| --- | --- | --- | --- |
| ONECUT1 | ONECUT1 | one cut homeobox 1 | SNV |
| HLA-DQB1 | HLA-DQB1 | major histocompatibility complex, class II, DQ beta 1 | SCNA |
| HLA-DRA | HLA-DRA | major histocompatibility complex, class II, DR alpha | SCNA |
| SPTA1 | SPTA1 | spectrin, alpha, erythrocytic 1 (elliptocytosis 2) | SNV |
| CTBS | CTBS | chitobiase, di-N-acetyl- | Transcript fusion |
| HLA-DQB2 | HLA-DQB2 | major histocompatibility complex, class II, DQ beta 2 | SCNA |
| TAP2 | TAP2 | transporter 2, ATP-binding cassette, sub-family B (MDR/TAP) | SCNA |
| GPR112 | GPR112 | G protein-coupled receptor 112 | SCNA |
| CASKIN1 | CASKIN1 | CASK interacting protein 1 | SNV |
| GPSM3 | GPSM3 | G-protein signaling modulator 3 | SCNA |
| SEL1L2 | SEL1L2 | sel-1 suppressor of lin-12-like 2 (C. elegans) | SNV |
| ZBTB34 | ZBTB34 | zinc finger and BTB domain containing 34 | SNV |
| APH1A | APH1A | anterior pharynx defective 1 homolog A (C. elegans) | SNV |
| HLA-DRB1 | HLA-DRB1 | major histocompatibility complex, class II, DR beta 1 | SCNA |
| NDRG1 | NDRG1 | N-myc downstream regulated 1 | SCNA |
| NOL6 | NOL6 | nucleolar protein family 6 (RNA-associated) | SNV |
| PBX2 | PBX2 | pre-B-cell leukemia homeobox 2 | SCNA |
| HLA-DQA2 | HLA-DQA2 | major histocompatibility complex, class II, DQ alpha 2 | SCNA |
| MYC | MYC | v-myc myelocytomatosis viral oncogene homolog (avian) | SCNA |
| WDR87 | WDR87 | WD repeat domain 87 | SNV |
| HLA-DRB5 | HLA-DRB5 | major histocompatibility complex, class II, DR beta 5 | SCNA |
| RNASEL | RNASEL | ribonuclease L (2',5'-oligoisoadenylate synthetase-dependent) | SNV |
| HLA-DOB | HLA-DOB | major histocompatibility complex, class II, DO beta | SCNA |
| TGFB2 | TGFB2 | transforming growth factor, beta 2 | SNV |
| GNG5 | GNG5 | guanine nucleotide binding protein (G protein), gamma 5 | Transcript fusion |
| FABP2 | FABP2 | fatty acid binding protein 2, intestinal | Transcript fusion |
| MAFA | MAFA | v-maf musculoaponeurotic fibrosarcoma oncogene homolog A (avian) | SNV |
| BTNL2 | BTNL2 | butyrophilin-like 2 (MHC class II associated) | SCNA |
| TP53 | TP53 | tumor protein p53 | SNV |
| MCM4 | MCM4 | minichromosome maintenance complex component 4 | Gene fusion |
| PEG10 | PEG10 | paternally expressed 10 | SNV |
| GPR124 | GPR124 | G protein-coupled receptor 124 | SNV |
| HLA-DQA1 | HLA-DQA1 | major histocompatibility complex, class II, DQ alpha 1 | SCNA |
| NOTCH4 | NOTCH4 | notch 4 | SCNA |
| MUC16 | MUC16 | mucin 16, cell surface associated | SCNA |
| SNTB1 | SNTB1 | syntrophin, beta 1 (dystrophin-associated protein A1, 59kDa, basic component 1) | Gene fusion |
| TNFSF15 | TNFSF15 | tumor necrosis factor (ligand) superfamily, member 15 | SNV |
| GZF1 | GZF1 | GDNF-inducible zinc finger protein 1 | SMV |
| FAT1 | FAT1 | FAT tumor suppressor homolog 1 (Drosophila) | Indel |
