# Supplementary material for: Characterization of genetic aberrations in a single case of metastatic thymic adenocarcinoma
Source: BMC Cancer. 2017 May 15;17:330. doi: 10.1186/s12885-017-3282-9 (PMC5432996; doi:10.1186/s12885-017-3282-9)
Supplement: Supplementary file 14 — Fifty-two GO terms enriched in the biological process (DOCX 20 kb) [file 12885_2017_3282_MOESM14_ESM.docx]

**Table S8. Fifty-two GO terms enriched in the biological process.**

| GO-ID | Adjusted  PValue_test | description_test | N_test | name | nn_test | pValue_test | X_test | xx_test |
| --- | --- | --- | --- | --- | --- | --- | --- | --- |
| 2495 | 4.59E-11 | antigen processing and presentation of peptide antigen via MHC class II | 38470 | 2495 | 95 | 5.91E-14 | 39 | 8 |
| 19886 | 4.59E-11 | antigen processing and presentation of exogenous peptide antigen via MHC class II | 38470 | 19886 | 93 | 4.96E-14 | 39 | 8 |
| 2504 | 1.69E-10 | antigen processing and presentation of peptide or polysaccharide antigen via MHC class II | 38470 | 2504 | 117 | 3.26E-13 | 39 | 8 |
| 34112 | 1.80E-10 | positive regulation of homotypic cell-cell adhesion | 38470 | 34112 | 210 | 6.66E-13 | 39 | 9 |
| 50870 | 1.80E-10 | positive regulation of T cell activation | 38470 | 50870 | 206 | 5.60E-13 | 39 | 9 |
| 1903039 | 1.80E-10 | positive regulation of leukocyte cell-cell adhesion | 38470 | 1903039 | 211 | 6.95E-13 | 39 | 9 |
| 31294 | 3.03E-10 | lymphocyte costimulation | 38470 | 31294 | 78 | 1.56E-12 | 39 | 7 |
| 31295 | 3.03E-10 | T cell costimulation | 38470 | 31295 | 77 | 1.42E-12 | 39 | 7 |
| 2478 | 3.68E-10 | antigen processing and presentation of exogenous peptide antigen | 38470 | 2478 | 239 | 2.14E-12 | 39 | 9 |
| 22409 | 3.91E-10 | positive regulation of cell-cell adhesion | 38470 | 22409 | 246 | 2.77E-12 | 39 | 9 |
| 19884 | 3.91E-10 | antigen processing and presentation of exogenous antigen | 38470 | 19884 | 246 | 2.77E-12 | 39 | 9 |
| 45785 | 4.75E-10 | positive regulation of cell adhesion | 38470 | 45785 | 378 | 3.68E-12 | 39 | 10 |
| 2376 | 8.11E-10 | immune system process | 38470 | 2376 | 2896 | 6.79E-12 | 39 | 19 |
| 51251 | 9.47E-10 | positive regulation of lymphocyte activation | 38470 | 51251 | 279 | 8.55E-12 | 39 | 9 |
| 48002 | 1.21E-09 | antigen processing and presentation of peptide antigen | 38470 | 48002 | 289 | 1.17E-11 | 39 | 9 |
| 50863 | 1.41E-09 | regulation of T cell activation | 38470 | 50863 | 296 | 1.45E-11 | 39 | 9 |
| 1903037 | 1.58E-09 | regulation of leukocyte cell-cell adhesion | 38470 | 1903037 | 304 | 1.84E-11 | 39 | 9 |
| 2696 | 1.58E-09 | positive regulation of leukocyte activation | 38470 | 2696 | 304 | 1.84E-11 | 39 | 9 |
| 50867 | 1.90E-09 | positive regulation of cell activation | 38470 | 50867 | 313 | 2.38E-11 | 39 | 9 |
| 34110 | 1.90E-09 | regulation of homotypic cell-cell adhesion | 38470 | 34110 | 314 | 2.45E-11 | 39 | 9 |
| 50852 | 3.18E-09 | T cell receptor signaling pathway | 38470 | 50852 | 124 | 4.30E-11 | 39 | 7 |
| 30155 | 3.50E-09 | regulation of cell adhesion | 38470 | 30155 | 677 | 4.96E-11 | 39 | 11 |
| 19882 | 3.53E-09 | antigen processing and presentation | 38470 | 19882 | 342 | 5.23E-11 | 39 | 9 |
| 7166 | 6.72E-09 | cell surface receptor signaling pathway | 38470 | 7166 | 3388 | 1.04E-10 | 39 | 19 |
| 60333 | 7.31E-09 | interferon-gamma-mediated signaling pathway | 38470 | 60333 | 143 | 1.18E-10 | 39 | 7 |
| 22407 | 9.98E-09 | regulation of cell-cell adhesion | 38470 | 22407 | 390 | 1.67E-10 | 39 | 9 |
| 51249 | 1.34E-08 | regulation of lymphocyte activation | 38470 | 51249 | 405 | 2.33E-10 | 39 | 9 |
| 50851 | 1.50E-08 | antigen receptor-mediated signaling pathway | 38470 | 50851 | 161 | 2.71E-10 | 39 | 7 |
| 6955 | 2.68E-08 | immune response | 38470 | 6955 | 2033 | 5.01E-10 | 39 | 15 |
| 2694 | 3.90E-08 | regulation of leukocyte activation | 38470 | 2694 | 463 | 7.55E-10 | 39 | 9 |
| 48522 | 4.66E-08 | positive regulation of cellular process | 38470 | 48522 | 5461 | 9.30E-10 | 39 | 22 |
| 71346 | 4.84E-08 | cellular response to interferon-gamma | 38470 | 71346 | 194 | 9.98E-10 | 39 | 7 |
| 2682 | 5.06E-08 | regulation of immune system process | 38470 | 2682 | 1791 | 1.08E-09 | 39 | 14 |
| 50865 | 6.07E-08 | regulation of cell activation | 38470 | 50865 | 494 | 1.33E-09 | 39 | 9 |
| 34341 | 8.48E-08 | response to interferon-gamma | 38470 | 34341 | 213 | 1.91E-09 | 39 | 7 |
| 2684 | 2.99E-07 | positive regulation of immune system process | 38470 | 2684 | 1085 | 6.94E-09 | 39 | 11 |
| 71310 | 4.09E-07 | cellular response to organic substance | 38470 | 71310 | 2527 | 9.75E-09 | 39 | 15 |
| 48518 | 4.60E-07 | positive regulation of biological process | 38470 | 48518 | 6864 | 1.13E-08 | 39 | 23 |
| 48583 | 4.70E-07 | regulation of response to stimulus | 38470 | 48583 | 4482 | 1.18E-08 | 39 | 19 |
| 70887 | 6.43E-07 | cellular response to chemical stimulus | 38470 | 70887 | 3070 | 1.66E-08 | 39 | 16 |
| 19221 | 8.19E-07 | cytokine-mediated signaling pathway | 38470 | 19221 | 682 | 2.16E-08 | 39 | 9 |
| 23052 | 1.35E-06 | signaling | 38470 | 23052 | 8728 | 3.75E-08 | 39 | 25 |
| 44700 | 1.35E-06 | single organism signaling | 38470 | 44700 | 8722 | 3.69E-08 | 39 | 25 |
| 7165 | 1.46E-06 | signal transduction | 38470 | 7165 | 8045 | 4.24E-08 | 39 | 24 |
| 10033 | 1.46E-06 | response to organic substance | 38470 | 10033 | 3278 | 4.18E-08 | 39 | 16 |
| 7154 | 2.94E-06 | cell communication | 38470 | 7154 | 9085 | 8.72E-08 | 39 | 25 |
| 71345 | 3.84E-06 | cellular response to cytokine stimulus | 38470 | 71345 | 831 | 1.16E-07 | 39 | 9 |
| 2768 | 6.60E-06 | immune response-regulating cell surface receptor signaling pathway | 38470 | 2768 | 634 | 2.04E-07 | 39 | 8 |
| 50776 | 7.71E-06 | regulation of immune response | 38470 | 50776 | 1205 | 2.43E-07 | 39 | 10 |
| 48584 | 7.72E-06 | positive regulation of response to stimulus | 38470 | 48584 | 2320 | 2.49E-07 | 39 | 13 |
| 2429 | 8.99E-06 | immune response-activating cell surface receptor signaling pathway | 38470 | 2429 | 445 | 2.95E-07 | 39 | 7 |
| 34097 | 9.96E-06 | response to cytokine | 38470 | 34097 | 942 | 3.34E-07 | 39 | 9 |
