# Supplementary material for: Characterization of genetic aberrations in a single case of metastatic thymic adenocarcinoma
Source: BMC Cancer. 2017 May 15;17:330. doi: 10.1186/s12885-017-3282-9 (PMC5432996; doi:10.1186/s12885-017-3282-9)
Supplement: Supplementary file 15 — Enriched KEGG and Reactome pathways (DOCX 14 kb) [file 12885_2017_3282_MOESM15_ESM.docx]

**Table S9. Enriched KEGG and Reactome pathways**

| Pathway Name | Pathway Id | Source Name | gene count | p-value | corrected p-value |
| --- | --- | --- | --- | --- | --- |
| Asthma | 2818 | KEGG | 6 | 3.33E-11 | 3.43E-09 |
| Graft-versus-host disease | 2807 | KEGG | 6 | 2.02E-10 | 4.17E-09 |
| Antigen processing and presentation | 493 | KEGG | 7 | 1.95E-10 | 5.03E-09 |
| Leishmaniasis | 10355 | KEGG | 7 | 1.95E-10 | 5.03E-09 |
| Translocation of ZAP-70 to Immunological synapse | 13070 | REACTOME | 5 | 2.96E-10 | 5.09E-09 |
| Phosphorylation of CD3 and TCR zeta chains | 13071 | REACTOME | 5 | 4.10E-10 | 5.28E-09 |
| Type I diabetes mellitus | 525 | KEGG | 6 | 3.89E-10 | 5.73E-09 |
| Allograft rejection | 2793 | KEGG | 6 | 1.42E-10 | 7.30E-09 |
| Intestinal immune network for IgA production | 8118 | KEGG | 6 | 8.05E-10 | 9.21E-09 |
| Autoimmune thyroid disease | 2799 | KEGG | 6 | 1.36E-09 | 1.27E-08 |
| PD-1 signaling | 13091 | REACTOME | 5 | 1.25E-09 | 1.29E-08 |
| Staphylococcus aureus infection | 10357 | KEGG | 6 | 1.95E-09 | 1.67E-08 |
| Viral myocarditis | 8123 | KEGG | 6 | 2.75E-09 | 2.18E-08 |
| Generation of second messenger molecules | 13069 | REACTOME | 5 | 7.96E-09 | 5.47E-08 |
| Toxoplasmosis | 10385 | KEGG | 7 | 7.67E-09 | 5.64E-08 |
| Phagosome | 10394 | KEGG | 7 | 4.22E-08 | 2.71E-07 |
| Downstream TCR signaling | 13068 | REACTOME | 5 | 4.99E-08 | 3.02E-07 |
| MHC class II antigen presentation | 13065 | REACTOME | 6 | 1.76E-07 | 1.01E-06 |
| TCR signaling | 17097 | REACTOME | 5 | 2.66E-07 | 1.44E-06 |
| Interferon gamma signaling | 13077 | REACTOME | 5 | 3.13E-07 | 1.61E-06 |
| Costimulation by the CD28 family | 13092 | REACTOME | 5 | 3.39E-07 | 1.66E-06 |
| Systemic lupus erythematosus | 2805 | KEGG | 6 | 3.75E-07 | 1.75E-06 |
| Cell adhesion molecules (CAMs) | 440 | KEGG | 6 | 8.24E-07 | 3.69E-06 |
| Interferon Signaling | 18059 | REACTOME | 6 | 1.42E-06 | 6.10E-06 |
