# Supplementary material for: Characterization of genetic aberrations in a single case of metastatic thymic adenocarcinoma
Source: BMC Cancer. 2017 May 15;17:330. doi: 10.1186/s12885-017-3282-9 (PMC5432996; doi:10.1186/s12885-017-3282-9)
Supplement: Supplementary file 12 — The CTBS-GNG5 fusion transcript by intergenic splicing. The GNG5 gene (blue) and CTBS gene (red) are consecutively located in chromosome 6 via the intergenic region (64 kb). The read-through transcript is transcribed from their DNA sequences without termination between CTBS and GNG5. The region from the tail of CTBS exon 6 to the head of GNG5 exon 3 is spliced out across the intergenic region by the intergenic splicing event. The transcript possesses a fusion sequence between CTBS exon 6 and GNG5 exon 3. The CTBS-GNG5 fusion sequence is supported by 76 reads with a uniform read distribution (PDF 57 kb) [file 12885_2017_3282_MOESM12_ESM.pdf]

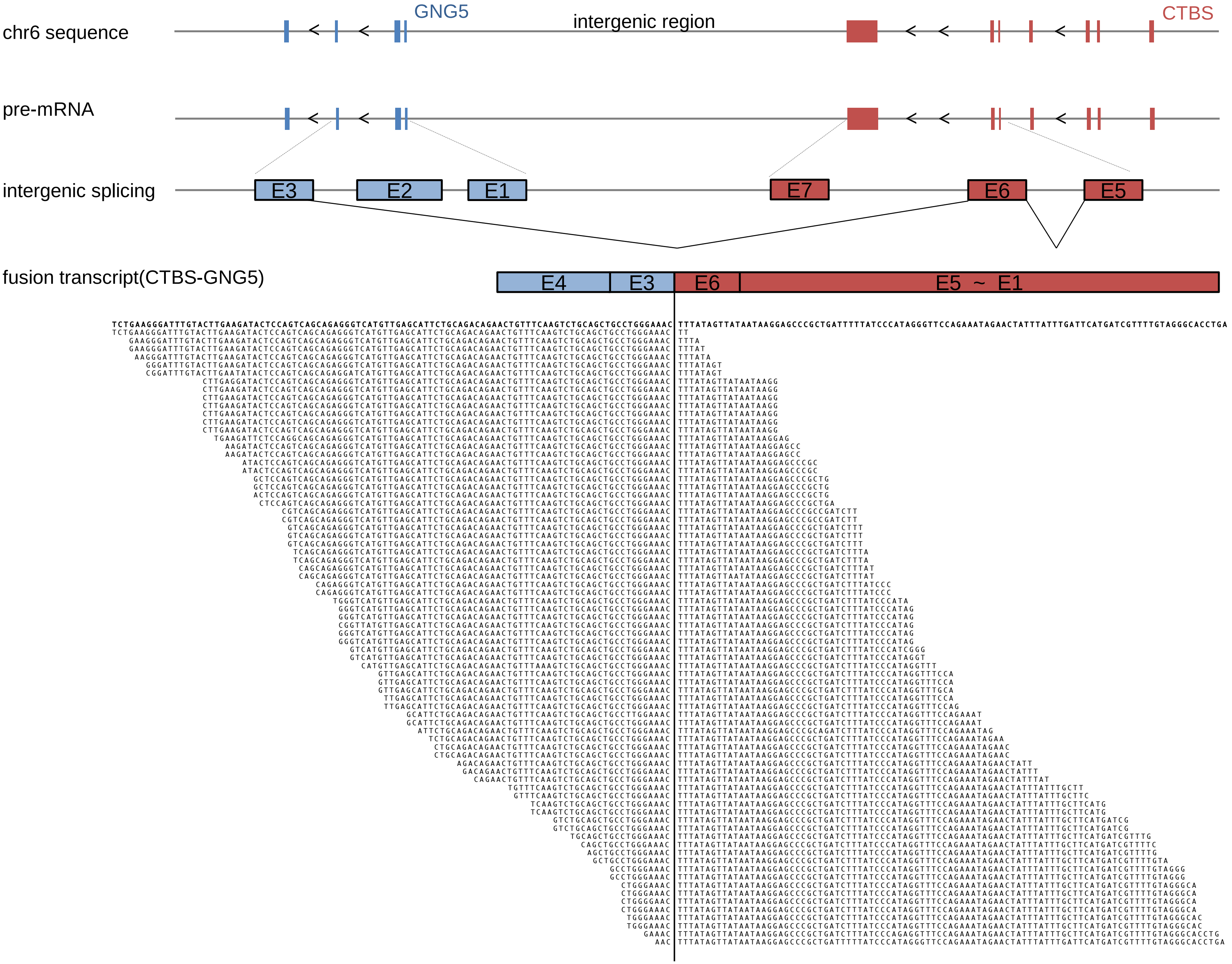

chr6 sequence
GNG5
intergenic region
CTBS
pre-mRNA
intergenic splicing
E3
E2
E1
E7
E6
E5
fusion transcript(CTBS-GNG5)
E4
E3
E6
E5 ~ E1
